# Effects of Nitrogen Application on Nitrogen Fixation in Common Bean Production

**DOI:** 10.3389/fpls.2020.01172

**Published:** 2020-08-06

**Authors:** Yarmilla Reinprecht, Lyndsay Schram, Frédéric Marsolais, Thomas H. Smith, Brett Hill, Karl Peter Pauls

**Affiliations:** ^1^ Department of Plant Agriculture, University of Guelph, Guelph, ON, Canada; ^2^ London Research and Development Centre, Agriculture and Agri-Food Canada, London, ON, Canada; ^3^ Lethbridge Research and Development Centre, Agriculture and Agri-Food Canada, Lethbridge, AB, Canada

**Keywords:** common bean, nitrogen, rhizobia, symbiotic nitrogen fixation, efficiency, yield

## Abstract

The nitrogen fixing ability of common bean (*Phaseolus vulgaris* L.) in association with rhizobia is often characterized as poor compared to other legumes, and nitrogen fertilizers are commonly used in bean production to achieve high yields, which in general inhibits nitrogen fixation. In addition, plants cannot take up all the nitrogen applied to the soil as a fertilizer leading to runoff and groundwater contamination. The overall objective of this work is to reduce use of nitrogen fertilizer in common bean production. This would be a major advance in profitability for the common bean industry in Canada and would significantly improve the ecological footprint of the crop. In the current work, 22 bean genotypes [including recombinant inbred lines (RILs) from the Mist × Sanilac population and a non-nodulating mutant (R99)] were screened for their capacity to fix atmospheric nitrogen under four nitrogen regimes. The genotypes were evaluated in replicated field trials on N-poor soils over three years for the percent nitrogen derived from atmosphere (%Ndfa), yield, and a number of yield-related traits. Bean genotypes differed for all analyzed traits, and the level of nitrogen significantly affected most of the traits, including %Ndfa and yield in all three years. In contrast, application of rhizobia significantly affected only few traits, and the effect was inconsistent among the years. Nitrogen application reduced symbiotic nitrogen fixation (SNF) to various degrees in different bean genotypes. This variation suggests that SNF in common bean can be improved through breeding and selection for the ability of bean genotypes to fix nitrogen in the presence of reduced fertilizer levels. Moreover, genotypes like RIL_38, RIL_119, and RIL_131, being both high yielding and good nitrogen fixers, have potential for simultaneous improvement of both traits. However, breeding advancement might be slow due to an inconsistent correlation between these traits.

## Introduction

Common (dry) bean (*Phaseolus vulgaris* L.) is the most important legume crop used for direct human consumption. It is low in fat but rich in proteins, slowly metabolizable starch, minerals, and fibers. Beans are staple foods in many regions of South America and Africa (Broughton et al., 2003). They grow well on medium to high-fertility soils with a minimum of 2% organic matter, neutral pH, high concentrations of phosphorus, calcium and magnesium, and low levels of aluminum and manganese (OMAFRA, 2017). Nitrogen availability is often a limiting factor for high productivity in bean crops. Therefore, nitrogen fertilizers are commonly applied to bean fields in countries where they are readily available. However, plants cannot utilize all the nitrogen fertilizer that is applied to the soil (Raun and Johnson, 1999; van Kessel and Hartley, 2000) leading to runoff and groundwater contamination. In symbiotic association with members of the Fabaceae (Leguminosae) family, the soil bacteria from the genus Rhizobia can fix atmospheric nitrogen (N_2_) and convert it to the usable NH_3_ form (Adams et al., 2016). Biological (symbiotic) nitrogen fixation (BNF, SNF) is a sustainable and economical alternative for supplying nitrogen to legume crops, including common bean and soybean (Thilakarathna and Raizada, 2018). It can reduce crop input costs by eliminating the need to purchase nitrogen fertilizer and reduce the negative impacts of unused nitrogen in the environment (Stevens et al., 2004; Good and Beatty, 2011; Khan et al., 2020).

The efficiency of the nitrogen fixing activity of the symbiotic association between bacteria and plants varies among legume species, and common bean is often characterized as poor nitrogen fixer (Isoi and Yoshida, 1991; Bliss, 1993; Hardarson et al., 1993; Martínez-Roméro, 2003; Hardarson, 2004; Peoples et al., 2009). However, significant variation in SNF potential exists among beans. In general, Mesoamerican indeterminate and climbing cultivars have better nodulation and higher SNF compared to the majority of Andean determinate (bush-type) cultivars (Wilker et al., 2019). Among market classes, pinto and navy beans fix more nitrogen compared to the black and kidney beans (Sanyal et al., 2018). A number of genotypes that fix substantial amounts of nitrogen (Miranda and Bliss, 1991), with the estimated mean value of 39% nitrogen derived from atmosphere (%Ndfa), have been identified across different environments (Peoples et al., 2009). Wilker et al. (2019) found considerable variation in nitrogen fixing capacity (measured as %Ndfa), ranging from 21 to 76%, in a collection of Mesoamerican and Andean bean genotypes. Estimates of these values are complicated by interactions among various factors, including genotype, rhizobial strain, growth environment, legume growth stage, and plant-available soil nitrogen (Unkovich and Pate, 2000; Wilker et al., 2019).

Although inoculations of common bean with rhizobia have resulted in increases in biomass, yield, and nitrogen levels in the plants in controlled environments (Saito, 1982), the rhizobium-*Phaseolus* symbiosis has been less efficient under field conditions (Graham, 1981; Buttery et al., 1987; Hungria and Vargas, 2000). Most modern bean cultivars were developed for their high yield potential in nitrogen-rich fertile soils, but the high fertility levels may suppress expression of desirable traits associated with nitrogen fixation. A negative correlation between SNF and the available soil nitrogen has been consistently identified in legumes (Kiers et al., 2003; Salvagiotti et al., 2008; Schipanski et al., 2010). In particular, nitrogen fixation is an energy demanding process (Burris, 2001; Mus et al., 2016) and less energy is needed to uptake mineral nitrogen than to fix nitrogen symbiotically (McKenzie et al., 2001).

However, there are differences in the nodulation sensitivity to nitrogen levels among legumes (Harper, 1984). A number of studies have shown that nodulation is inhibited in soybean by nitrogen levels exceeding 5 mM (Saito et al., 2014). In a recent study, evaluating 16 common bean genotypes from different market classes under four nitrogen treatments [not inoculated low N (30 kg ha^−1^) and high N (100 kg ha^−1^) and two rhizobia strains], Akter et al. (2018) confirmed inhibition of nitrogen fixation by a high dose of nitrogen fertilizer. Genotype-specific sensitivity of nitrogen fixation to mineral nitrogen was identified in common bean (Hardarson, 1993; Farid et al., 2016). Nitrogen concentrations of 2.5 and 5 mM had positive effects on SNF in beans (Jiang et al., 2020). Similarly, in controlled greenhouse and growth chamber experiments, Gan et al. (2004) demonstrated that low concentrations (1 and 3.75 mM, kept constant) of inorganic nitrogen increase SNF in soybean. The selection for improved SNF under low soil nitrogen has been proposed.

In addition to the genetic background, environmental factors such as drought (Kumarasinghe et al., 1992), salinity (Bouhmouch et al., 2005; Faghire et al., 2011), and soil deficiencies in phosphorus, potassium, and sulfur (Tsai et al., 1993; Divito and Sadras, 2014) can greatly influence legume SNF capability. Sensitivity to moisture stress and requirements for high amounts of photosynthates and phosphorous can reduce rhizobia activity. The interaction of these factors and the environment reduces the capability of most common bean cultivars to fix nitrogen in the tropics and subtropics (Park and Buttery, 1989; Fageria et al., 2014). Some of the factors that contribute to the low nitrogen fixation observed in common bean include delayed nodule formation, insufficient nodule mass, and ineffectiveness of nodules formed on root system (Isoi and Yoshida, 1991).

The nitrogen fixing ability of legumes can be analyzed using various methods and different tissues. In general, the amount of fixed nitrogen depends on the method and/or reference crop (cultivar) used for its determination (Unkovich and Pate, 2001; Unkovich et al., 2008). The ^15^N enriched isotope dilution and the ^15^N natural abundance methods have been widely used for SNF determination. In the isotope dilution technique, enriched ^15^N-labeled fertilizer is applied to both legume and reference crops, while in the natural abundance method the naturally occurring difference in atom% ^15^N of soil available nitrogen and atmospheric nitrogen is used to estimate SNF. In both methods, differences in dilution of ^15^N between the legume and the reference crop are used to calculate SNF. Although it is considered as the most accurate method for estimating the amount of nitrogen derived from nitrogen fixation and nitrogen from fertilizer and soil, the ^15^N dilution method has some limitations, including a requirement for accessing labeled fertilizer and the difficulty in establishing a stable and uniform ^15^N enrichment of soil mineral nitrogen in space and time. Alternatively, the ^15^N abundance method can be easily utilized in field settings but analyses, performed either by GC–MS or optical emission spectrometry, may be expensive.

Significant positive correlations identified between %Ndfa in seeds and shoots of bush (Polania et al., 2016b) and climbing genotypes (Barbosa et al., 2018) suggested that the SNF ability of common bean can be quantified from the seeds. Common beans essentially translocate much of their nitrogen from vegetative parts to the seeds (Lynch and White, 1992; Ramaekers et al., 2013) making the ^15^N natural abundance assessment method applied to mature seeds a convenient way of comparing the relative capacities of genotypes to fix nitrogen during a growing season (Wilker et al., 2019).

The existence of genetic variability among beans indicates that the traits associated with SNF could be improved through breeding (Bliss, 1993; Farid and Navabi, 2015). However, inconsistent or no association between %Ndfa and yield (Farid et al., 2017) indicates that the improvement could be slow. Indices derived from yield measurements could simplify selection of bean genotypes with good nitrogen fixing capabilities. For example, the SNF relative efficiency index (SNFI) developed by Farid (2015) distinguishes between bean genotypes with the same yielding response to nitrogen application and rhizobia inoculation (SNFI = 0), better yielding response to inoculation than to nitrogen application (SNFI > 0) and better yielding response of genotypes to nitrogen application than to inoculation (SNFI < 0). In addition, a number of quantitative trait loci (QTL) for SNF-related traits were identified in bean linkage and association mapping studies. Some of these genomic regions contain potential candidate genes that could accelerate SNF-related breeding in bean, which was slow due to the complex genetic architecture of SNF. The trait is controlled by a number of genes with small effects and significantly influenced by the environment (Kamfwa et al., 2019).

Recent work by our group at Guelph has identified bean cultivars and breeding lines with greater potential for nitrogen fixation (Farid and Navabi, 2015). The objectives of the current research were to measure the variation in the sensitivity of nitrogen fixation to inhibition by nitrogen fertilizer in a set of beans selected from the previous work and to identify genotypes that may be useful starting point in breeding for highly productive bean cultivars under reduced, but not zero, nitrogen fertilizer regimes. Data obtained from a three-year study allowed a detailed analysis of the effects of bean genotype, nitrogen fertilizer and rhizobia on SNF (calculated as %Ndfa) to be conducted and its relationships with yield and a number of yield-related characteristics to be made.

## Material and Methods

### Plant Material

**Figure 1:**
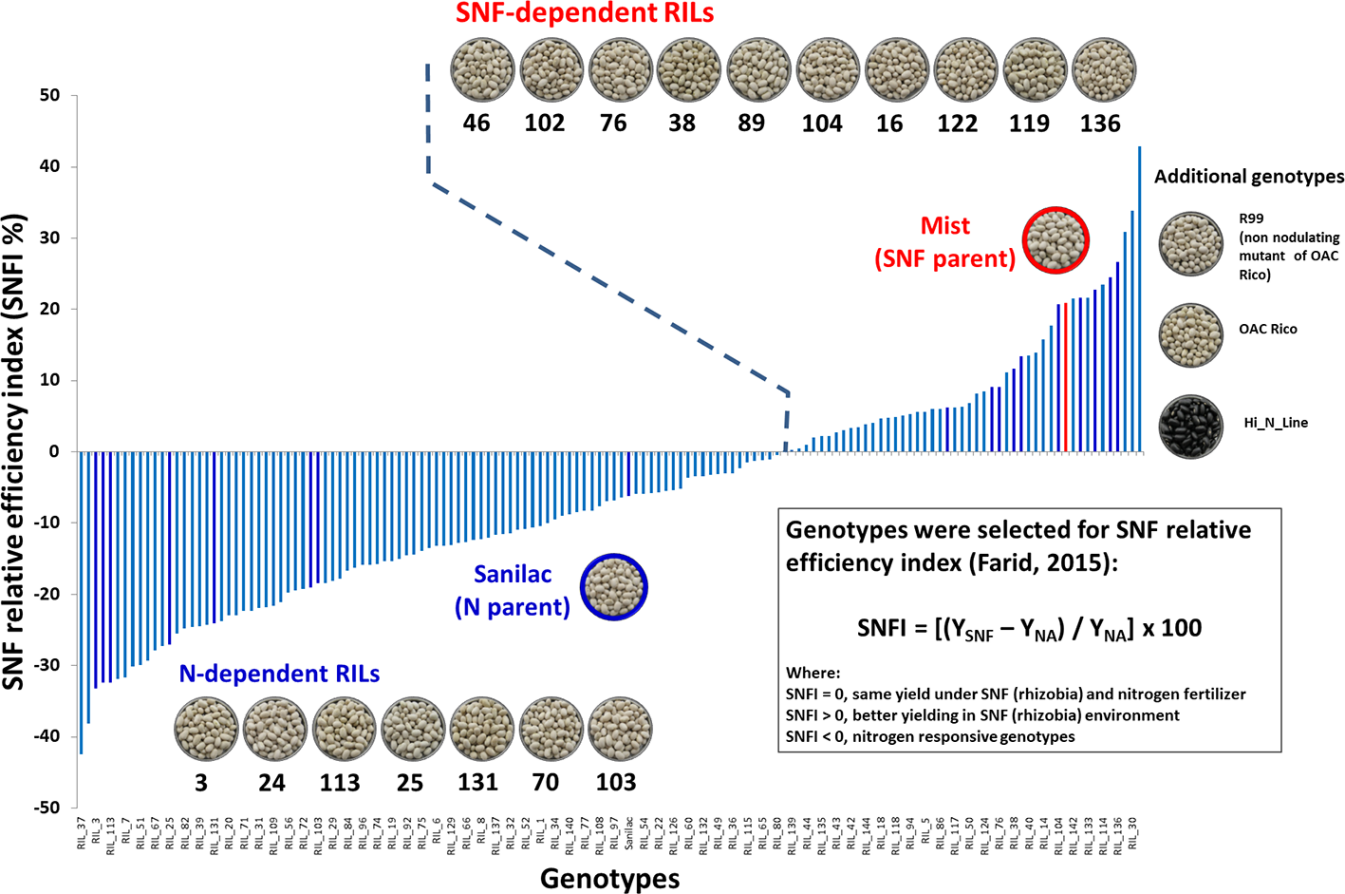
Selection of the common bean genotypes for nitrogen study. Seventeen recombinant inbred lines (RILs) were selected from a Mist x Sanilac mapping population developed at the University of Guelph, ON. The selection was based on the symbiotic nitrogen fixation efficiency index {SNFI = [(Y_SNF_ – Y_NA_) / Y_NA_] x 100} and included 10 SNF-dependent RILs (high yield under SNF condition) and seven N-dependent RILs (high yield under nitrogen application). Parental genotypes, Mist (SNF parent) and Sanilac (N parent), were also included. Additional genotypes (right): R99 is a non nodulating mutant of cultivar OAC Rico; OAC Rico (wild type); Hi_N_Line, black genotype selected for high %Ndfa. SNFI-based genotype distribution was modified with permission (Farid, 2015; PhD thesis, University of Guelph).

The work was conducted in two experiments over three years. In 2016, a nitrogen study was initiated with 20 small seeded [Middle American (Mesoamerican) gene pool] common bean genotypes, including 17 recombinant inbred lines (RILs) from a Mist × Sanilac mapping population, two parental genotypes (Sanilac and Mist) and a non-nodulating line R99 (**Figure 1**). The aim of the study was to verify variability of the beans to fix nitrogen in N-poor soil that was identified previously in the whole population. The selection of RILs was based on the SNFI index (Farid, 2015) and included 10 SNF-dependent RILs [high yield under SNF condition (RIL_16, RIL_38, RIL_46, RIL_76, RIL_89, RIL_102, RIL_104, RIL_119, RIL_122 and RIL_136)] and seven N-dependent RILs [high yield under nitrogen application (RIL_3, RIL_24, RIL_25, RIL_70, RIL103, RIL_113 and RIL_131)]. Mist (SNF parent, high yield under SNF condition) is an upright indeterminate (type II), high-yielding, late maturing navy bean cultivar resistant to common bacterial blight (CBB) and bean common mosaic virus (BCMV) races 1 and 15. The cultivar was derived from the modified 8-way cross (spscbbr136/PI207262//ICB-10/Vax4///OAC Speedvale/Avanti//OAC 99-1/OAC Rex; University of Guelph), tested as ACUG 10-6 and registered in 2013 (Khanal et al., 2017). Sanilac (N parent, high yield under application of nitrogen fertilizer) was developed at the Michigan State University as an x-ray induced mutant of cultivar Michelite and released in 1956 (Kelly, 2010). It is a determinate-bush, early maturing navy bean, “significant for its upright bush habit, earlier maturity, anthracnose and avoidance of white mold” (Kelly, 1999) but, characterized as a low-nodulating, poor nitrogen fixing genotype (Bliss, 1993). R99 is a non-nodulating mutant of cultivar OAC Rico developed at Agriculture and Agri-Food Canada Greenhouse and Crops Processing Centre at Harrow, ON (Park and Buttery, 1997) and registered as a genetic stock in 2006 (Park and Buttery, 2006). It was used as a reference genotype for estimating nitrogen fixation measured as %Ndfa in the current study.

Two additional genotypes were included in the 2017 and 2018 trials. OAC Rico is a backcross-derived anthracnose-resistant cultivar developed at University of Guelph from the cross Ex Rico 23 × Narda, tested as OAC 81-4 and registered in 1983. It is determinate cultivar similar in agronomic performance to Ex Rico 23 (Beversdorf, 1984). Hi_N_Line is the only black genotype tested in the study and was included because of its high %Ndfa value determined by a seed assay (Wilker et al., 2019).

### Field Evaluation

Field trials were conducted on nitrogen depleted (N-poor) soils (three adjacent fields) at the University of Guelph Elora research station {ERS, latitude 43^°^39′N, longitude 80^°^25′W, elevation 376.4 m; 2,680 CHU [Crop Heat Unit; it is a heat unit system used to provide an estimate of the average accumulated daily temperature available for crop growth in a region (Brown and Bootsma, 1993)]}, ON, Canada in three years (2016–2018). Soil at the ERS is an imperfectly-drained undulating (1 to 5% slope) loam [London loam series Grey Brown Luvisolic (Typic Hapludalf) soil]. The N-poor soils were created by planting barley without nitrogen fertilizer and complete removal of the immature crop from the field, in two consecutive years.

Experiments were set in a split-split-plot design (SSPD) with four replications (same field). Within each replication, randomization was done at three levels: main plot (randomization 1), sub plot (randomization 2) and sub sub plot (randomization 3). In 2016 (experiment 1, field 1), the main plot was nitrogen [two levels: no nitrogen (NoN) and 100 kg ha^−1^ (N)], the sub plot was rhizobia [two levels: with (R) and without rhizobia (NoR)], and the genotype (20) was set as a sub sub plot. To simplify field operations, rhizobium was set as a main plot and nitrogen as a sub plot in the 2017 (field 2) and 2018 (field 3) trials (experiment 2). In addition, two new genotypes (OAC Rico and Hi_N_Line) were included, and 22 genotypes were evaluated in the second experiment. Trials were machine planted in 4-row, 6-m-long plots with 36-cm row spacing and 350 seeds per plot (1 June 2016, 13 June 2017, and 11 June 2018, respectively).

For the nitrogen treatment (N), 100 kg ha^−1^ N (granular urea, 46-0-0) was applied the same day after planting. For the rhizobia treatment (R), a combination of *Rhizobium leguminosarum* biovar viceae, *Rhizobium leguminosarum* biovar phaseoli and *Bradyrhizobium* sp. (McKenzie Seeds Garden Inoculant for Peas, Snap and Lima Beans - Vesey's Seeds Ltd., York, PEI, Canada) was used. A peat-based powder was applied to bare seed in each seed plot envelope 1 h before planting at the recommended rate (42 g per 2.3 kg of seed).

P-K fertilizers (200 kg ha^−1^ of 0-20-20) were applied before planting (late May in each experimental year). All herbicides and pesticides were applied with a tractor-mounted sprayer. The pre plant incorporated tank mix including: Pursuit® (BASF Canada) at 220 ml ha^−1^, Dual II Magnum® (Syngenta Canada) at 1.75 L ha^−1^ and Frontier® Maxx (BASF Canada) at 0.75 L ha^−1^ was applied in late May. In 2018 (4 July), the additional post emergent tank mix spray consisting of Basagran® (BASF Canada) at 2.25 L ha^−1^, Excel® Super (Bayer Canada) at 0.67 L ha^−1^, and Assist® (BASF Canada) at 1 L ha^−1^ was applied. The post planting pesticides application varied among the three years. In 2016 (July 12) a single tank mix of Admire® (Bayer Canada) at 0.2 L ha^−1^, Headline® (BASF) at 0.4 L ha^−1^ and Allegro® (Syngenta Canada) at 1 L ha^−1^ was applied. However, three pesticide applications [July 5: tank mix of Lagon® (Loveland Canada) at 1 L ha^−1^, Headline® at 0.4 L ha^−1^, Allegro® at 1 L ha^−1^ and Matador® (Syngenta Canada) at 40 ml ha^−1^; July 18: tank mix of Lagon® at 1 L ha^−1^, Quadris® (Syngenta Canada) at 0.5 L ha^−1^, Allegro® at 1 L ha^−1^ and Matador® at 40 ml ha^−1^; August 10: tank mix of Headline® at 0.4 L ha^−1^ and Allegro® at 1 L ha^−1^] in 2017 and two applications in 2018 [July 12: tank mix of Quadris® at 0.5 L ha^−1^, Allegro® at 1 L ha^−1^, Cygon® (FMC Canada) at 1 L ha^−1^ and Matador® at 40 ml ha^−1^. August 9: tank mix of Allegro® at 1 L ha^−1^ and Quadris® at 0.5 L ha^−1^] were necessary to control pests/diseases in the bean fields.

The fields were kept free of weeds, and when necessary, additional weeding was performed by cultivating weeds between the rows and manually within the rows by hawing or pulling weeds. The plots were combine-harvested (19 September 2016, 22 October 2017, and 12 October 2018).

### Weather Conditions at ERS in Bean Growing Seasons

Historical weather data, daily weather data at ERS for the three experimental years (2016, 2017, and 2018), and Canadian climate normals and averages data for the 1981–2010 period were collected from the nearest station (Fergus Shand Dam, ON; 43°44′05.088″ N, 80°19′49.098″ W, elevation 417.6 m; meets WMO standards for temperature and precipitation) (available at: http://climate.weather.gc.ca/climateData/dailydata_e.html and/climate_normals/) (**Supplementary Figure S1**).

### Soil Analysis

Prior to planting, a standard soil sample probe (Oakfield Classic Soil Probe) was used to randomly collect 10–16 soil samples at 0–9 inch (22.86 cm) depths across all areas of each nitrogen treatment (N and NoN) within each replication to create a bulk sample. After hand mixing, representative samples were sent for analysis to the SGS Agri-Food Laboratories Inc., Guelph, ON. The soil was analyzed only in 2017 and 2018 trials and did not include testing for the presence of indigenous rhizobia.

### Data Collection

In the 2016 pilot study (experiment 1), plot-based above ground crop data were collected for five traits, including maturity, harvestability, yield, carbon isotope discrimination, and percent nitrogen derived from atmosphere in seeds. Four additional traits (flowering, plant height, seed weight, and leaf chlorophyll content) were evaluated in experiment 2 conducted in 2017 and 2018 (nine traits in total).

#### Phenological Traits

Days to flowering (DF, flowering) was reported as a number of days from planting to 50% of plants in a plot with at least one fully opened flower. Days to maturity (DM, maturity) was calculated as a number of days from planting to physiological maturity.

#### Yield and Yield-Related Traits

Yield (YD) was calculated from the seed weight measured for each plot and adjusted to 18% moisture content (kg ha^−1^). Hundred seed weight (SW) was the weight of 100 threshed seeds, expressed in g and adjusted to 18% moisture content. Plant height (PH) was measured from the soil surface to the tip of the main stem at the mid-pod filling stage and expressed in cm. Harvestability (HR) was determined at maturity as a suitability (standability) of plants in plot for mechanical harvesting using a scale of 1 to 5 [where 1 represents erect plants suitable for combine harvesting) and 5 are prostrate plants (not suitable for machine harvesting)].

Relative leaf chlorophyll content (SPAD) was measured as greenness of the plants using a portable non-destructive SPAD (Soil Plant Analysis Development) chlorophyll meter (SPAD-502, Minolta, Japan) on eight fully expanded young leaves in a plot (one plant in each of the four replication, with a single reading from two leaves) at mid-pod filling stage [63 days after planting (DAP)] and expressed as SPAD values (Monje and Bugbee, 1992).

#### Seed Isotope Analysis

SNF was estimated as percent nitrogen derived from atmosphere (%Ndfa) in seed samples using the ^15^N natural abundance method (Shearer and Kohl, 1986) and R99 (non-nodulating mutant of cultivar OAC Rico) as the reference genotype. Briefly, bean samples (25 seeds) were dried in a forced-air oven at 60^°^C for 48 h and ground to a fine powder. Approximately 5 mg of ground bean samples and standards (alfalfa and USGS40) was weighed into tin capsules (8 × 5 mm, Isomass Scientific Inc., Calgary, AB). The closed and compressed capsules were placed in 96-well microplates and sent to the Agriculture and Agri-Food Canada, Lethbridge Research Centre (Lethbridge, AB, Canada) for analysis. Data were collected for δ^15^N, total N and δ^13^C using a Finnigan Delta V Plus (Thermo Electron, Bremen, Germany) Isotope Ratio Mass Spectrometer (IRMS) equipped with a Flash 2000 Elemental Analyzer (Thermo Fisher Scientific, Voltaweg, Netherlands) and Conflo IV (Thermo Fisher Scientific, Bremen, Germany) interface between the elemental analyzer and the IRMS.

The %Ndfa values were calculated using the equation:

$$\%\text{Ndfa}=[(\delta^{15}{\text{N}}_{\text{ref. plant}}-\delta^{15}{\text{N}}_{\text{fix. plant}})/(\delta^{15}{\text{N}}_{\text{ref. plant}}-B)]\times 100$$

where:

δ^15^N_ref.plant_ is the d15N in a non-nodulating reference genotype (R99); to reduce impact of any natural ^15^N variation in soil, treatment-based d15N values for R99 were used in all calculations; δ^15^N_fix.plant_ is the d15N in the analyzed common bean samples.

B is the d15N of the beans grown in a nitrogen-free medium (entire nitrogen was obtained from the nitrogen fixation); B = −1.988 (Farid, 2015).

Carbon isotope discrimination (δ^13^C, ‰), as an indicator of water use efficiency (Farquhar et al., 1989), was obtained using the same seed samples taken for δ^15^N and was calculated using the following formula:

$$\delta^{13}C\;(‰)=(\delta_{a}-\delta_{p})/[1+(\delta_{p}/1000)]$$

where:

δ_a_ is the isotopic ratio of ^13^C to ^12^C (−8‰) in the atmospheric air and δ_p_ is isotopic ratio of ^13^C to ^12^C in the seed sample.

Abbreviations will be used only for the trait names consisting of more than three words (%Ndfa, δ^13^C and SPAD).

### Data Analysis

Data were analyzed using the generalized linear mixed models (Glimmix) procedure in SAS (Statistical Analysis System) v.9.4 software (SAS Institute, 2013). Treatments (genotypes, rhizobia, and nitrogen) were considered as fixed effects. Replication and interactions between replications and rhizobia and among replications, rhizobia, and nitrogen were evaluated as random effects. The following model was used to analyze data from the single experiment of the current study set as the split-split plot design (SSPD):

$${\text{Y}}_{\text{ijkl}}=\text{μ}+{\mathrm{Rep}}_{\text{i}}+{\text{R}}_{\text{j}}+\text{Rep}{\text{R}}_{\text{ij}}+{\text{N}}_{\text{k}}+\text{R}{\text{N}}_{\text{jk}}+\text{RepR}{\text{N}}_{\text{ijk}}+{\text{G}}_{\text{l}}+\text{R}{\text{G}}_{\text{jl}}+\text{N}{\text{G}}_{\text{kl}}+\text{RN}{\text{G}}_{\text{jkl}}+{\text{E}}_{\text{ijkl}}$$

where:

Y_ijkl_ = measurement of outcome variable

µ = overall mean

Rep_i_ = random effect of replication (Rep)

R_j_ = fixed effect of factor R (rhizobia)–main plot

RepR_ij_ = random interaction between the Rep and the factor R (main plot factor) –error term for factor R (main plot error)

N_k_ = fixed effect of factor N (nitrogen) **–** sub plot

RN_jk_ = fixed interaction between factor R and factor N

RepRN_ijk_ = random interaction between the Rep and the factor N (sub plot factor) and the factor R (main plot factor) –error term for factor N and the interaction between factors R (main plot factor) and N (sub plot error)

G_l_ = fixed effect of factor G (genotype)–sub sub plot

RG_jl_ = fixed interaction between factor R (rhizobia) and factor G (genotype)

NG_kl_ = fixed interaction between factor N (nitrogen) and factor G (genotype)

RNG_jkl_ = fixed interaction between factor R (rhizobia), factor N (nitrogen) and factor G (genotype)

E_ijkl_ = residual error (correct error term for sub sub plot factor G, RG, NG, and RNG)

For experiment 2, a combined analysis over two years (2017 and 2018) was also computed. In the combined analysis, year, replication (year), and interactions between replications and rhizobia and among replications, rhizobia and nitrogen were evaluated as random effects using ‘random’ and ‘covtest’ statements. When necessary, ‘nobound’ option was used to remove boundary constraints on covariances (0), which allows their estimates to be negative. A few outliers were removed, and data were re-analyzed. Line R99 was used as a non-nodulating reference genotype in %Ndfa calculations and was removed from the analysis of that trait. If significant treatment differences were detected, pairwise multiple comparisons (LSmeans) were made using a Tukey–Kramer method (for unequal sample sizes). Residual analysis diagnostic plots were used to check assumptions for Gaussian distribution (normality, homogeneity of error variances) and find “the most suitable remedy in case, violations were identified” (Kozak and Piepho, 2017).

The relationships among traits (raw data and LSmeans) were analyzed by Pearson’s correlation in SAS. Similar to Diaz et al. (2017), all four replications were used in the initial correlation analysis with the raw data. For each trait, correlations between treatments (C, R, N and RN) were also calculated. The identification of a number of significant relationships among the traits suggested that multivariate (principal component, PCA) analysis (LSmeans) would be a good method to reduce data complexity. After calculation (princomp in SAS), trait eigenvalues and cultivar scores were exported to Microsoft Excel to produce Genotype × Traits (GT) biplots.

To identify high yielding genotypes with good nitrogen fixing capabilities, yield and %Ndfa data were plotted against each other for each treatment and experiment [as described by Nyikako et al. (2014)]. Within each treatment, plots were divided into four quadrants based on the yield and %Ndfa mean values for that treatment. High yielding genotypes with good nitrogen fixing capabilities were identified in upper right quadrant.

Yield and %Ndfa values were also used to rank common bean genotypes. Ranking was performed at the experiment level, for each treatment (C, R, N and RN) and as a treatment sum (C + R + N + RN) for each experiment. Genotype rank was calculated as a sum of %Ndfa rank and YD rank (genotype rank = %Ndfa rank + YD rank).

## Results

### Soil Properties and Environmental Conditions

The study was initiated in 2016 as a pilot experiment without soil analysis. Plot soils were analyzed before planting the studies in 2017 and 2018, separately for the two nitrogen treatments [NoN(0) and N (100 kg ha^−1^)]. The nitrate levels in the N samples were higher than those measured for the NoN samples in both years; 2.4-fold in 2017 and 1.9-fold in 2018, respectively (**Supplementary Table S1**). Ammonium contents were very similar in the two samples (NoN and N) in 2017, but in 2018 they were 2.6-fold higher in the N soils. All of the other parameters had very similar values for the NoN and N soils in both years.

The beans were in the field 101 days in 2016, 131 days in 2017, and 123 days in 2018, respectively. During the bean growing season (June–September/October), the weather conditions at ERS varied considerably in three experimental years (**Supplementary Figure S1**). Year 2016 received the most rainfall (385 mm), while 2017 (277 mm) and 2018 (211 mm) were drier than the 30-year precipitation average for this site (363 mm; http://climate.weather.gc.ca/climateData/dailydata_e.html and/climate_normals/), and they can be considered to have been drought-stress environments (Farid and Navabi, 2015). Peaks of precipitation were observed in July (102 mm) and August (153 mm) in 2016 and in June 2017 (with 118 mm). The year 2018 was the driest and the warmest among the three years.

### Performance of Selected Bean Genotypes in N-poor Soils

#### Experiment 1: Feasibility of Growing Beans in N-Poor Soil

An initial study conducted with a selected set of 20 bean genotypes confirmed variability of nitrogen fixation in N-poor soil that was identified previously with a whole Mist × Sanilac population. Genotypes were significantly different for all five analyzed traits, including %Ndfa and yield (**Table 1** and **Supplementary Table S2**). The effect of nitrogen fertilizer was significant for maturity and δ^13^C, while rhizobia did not affect any of the traits. Few interactions that were identified [rhizobia*genotype (R*G) for harvestability and nitrogen*genotype (N*G) and R*N*G, respectively for %Ndfa] indicated that genotypes reacted differently (%Ndfa and harvestability) to four nitrogen/rhizobia treatments in 2016. Beans under the R treatment had significantly higher values for %Ndfa (41.5 *vs.* 18.0%) but yielded less and had higher δ^13^C values (smaller value indicates better water use efficiency) compared to the beans under the N treatment. The addition of mineral nitrogen, alone (N treatment) or in combination with rhizobia (RN treatment), as well as rhizobia inoculation (R treatment) significantly increased harvestability (lower value indicates better standability) compared to the control (**Table 2**).

**Table 1:** Type III tests of fixed effects from restricted likelihood analysis of split-split plot experiments with 20 common bean genotypes conducted at the Elora research station (ON, Canada) on created K-P land in 2016.

Traits^1^	Effect	Numerator DF	Denominator DF	F Value	Pr > F
%Ndfa^NB2^	Nitrogen (N)	1	3.001	6.35	0.0862
	Rhizobia (R)	1	5.967	5.02	0.0666
	N*R	1	5.967	0.37	0.5671
	Genotype (G)	18	201	4.60	<.0001
	N*G	18	201	4.07	<.0001
	R*G	18	201	0.77	0.7282
	N*R*G	18	201	1.81	0.0261
YD^NB^	Nitrogen (N)	1	3	5.41	0.1024
	Rhizobia (R)	1	6	1.05	0.3453
	N*R	1	6	0.16	0.7069
	Genotype (G)	19	228	22.62	<.0001
	N*G	19	228	1.37	0.1450
	R*G	19	228	0.40	0.9890
	N*R*G	19	228	1.09	0.3636
δ^13^C^NB^	Nitrogen (N)	1	3.008	28.08	0.0130
	Rhizobia (R)	1	6.014	0.02	0.8839
	N*R	1	6.014	0.34	0.5822
	Genotype (G)	19	226.1	15.52	<.0001
	N*G	19	226.1	0.97	0.5035
	R*G	19	226.1	1.33	0.1667
	N*R*G	19	226.1	1.49	0.0902
DM^NB^	Nitrogen (N)	1	2.966	10.68	0.0476
	Rhizobia (R)	1	6.003	1.57	0.2566
	N*R	1	6.003	0.10	0.7594
	Genotype (G)	19	224.1	114.28	<.0001
	N*G	19	224.1	1.21	0.2478
	R*G	19	224.1	0.91	0.5713
	N*R*G	19	224.1	0.66	0.8569
HR^NB^	Nitrogen (N)	1	3.069	3.73	0.1468
	Rhizobia (R)	1	6.01	5.11	0.0644
	N*R	1	6.01	0.20	0.6678
	Genotype (G)	19	225.1	17.71	<.0001
	N*G	19	225.1	1.36	0.1463
	R*G	19	225.1	2.53	0.0007
	N*R*G	19	225.1	1.61	0.0559

1 Traits analyzed in 2016: %Ndfa, percent nitrogen derived from atmosphere (%); YD, yield (kg ha^−1^); δ^13^C, carbon isotope discrimination (‰); DM, maturity (days); HR, harvestability (scale 1 to 5). Non-nodulating genotype R99 was removed from the %Ndfa analysis.

2 Glimmix procedure, 2016; Normal distribution, ddfm = kr (NB, nobound option).

**Table 2:** Treatment means for five traits evaluated in 20 common bean genotypes at the Elora research station, ON in 2016 pilot study.

Trait^1^	Treatment^2^				SEM^3^
	C	R	N	RN	
%Ndfa (%)	34.8	41.5	18.0	21.9	4.67/4.68
Yield (kg ha^−1^)	2941	2820	3084	3030	80.9
δ^13^C (‰)	20.4	20.3	20.0	20.0	0.06
Maturity (days)	100.1	100.6	101.3	102.2	0.55
Harvestability (score 1 to 5)	3.06	3.33	3.21	3.39	0.148

Least Squares means (LSmeans) and Standard Error of the Mean are presented (SEM) (Alpha = 0.05).

1 %Ndfa, percent nitrogen derived from atmosphere (non-nodulating genotype R99 was used as a reference in %Ndfa calculation and was excluded from the trait analysis); δ^13^C, carbon isotope discrimination; SPAD, leaf chlorophyll content.

2 C, control (NoR_NoN); R, rhizobia (R_NoN); N, nitrogen (NoR_N); RN, rhizobia + nitrogen (R_N).

3 SEM values were different for treatments with unbalanced data in %Ndfa (shown underlined).

There were significant differences among genotypes for %Ndfa, yield and yield-associated traits, both at the experiment level (**Table 3**) as well as the levels of individual treatments (**Supplementary Table S3**). At the experiment level (all treatments), RIL_119 had the highest value of %Ndfa (33.8%) but was late maturing (108 days, latest among all genotypes), and had a yield of 2,650 kg ha^−1^, which was 26% lower compared to the most productive line RIL_38 (3,582 kg ha^−1^)] (**Table 3**). However, in the presence of nitrogen fertilizer its capacity to fix nitrogen was reduced to 54% (control treatment 42.5 *vs.* 19.4% N treatment). In comparison, RIL_46 was the best nitrogen fixer under the N treatment; it had the highest %Ndfa (23.2%) and relatively high yield (2,975 kg ha^−1^). Cultivar Sanilac also had a high value for %Ndfa (23.2%) but had the lowest yield (2,342 kg ha^−1^) among the genotypes in 2016 (**Supplementary Table S3**).

**Table 3:** Performance of 20 common bean genotypes evaluated at the Elora research station, ON in 2016 pilot study.

Genotype	Characteristics evaluated^1^				
	%Ndfa	YD	δ^13^C	DM	HR
Mist	27.4 ab^2^	3,541 a	19.8 de	102.2 cd	2.22 i
Sanilac	27.4 ab	2,163 j	19.9 cde	87.8 g	2.75 ghi
RIL_3	27.2 ab	3,067 b–g	19.8 de	91.4 f	3.12 efg
RIL_16	28.6 a	2,755 fgh	20.1 b–e	105.8 ab	3.13 efg
RIL_24	28.9 a	2,828 fg	19.8 de	92.7 f	3.06 efg
RIL_25	27.3 ab	2,946 c–g	20.6 a	101.4 d	3.63 b–e
TIL_38	33.7 a	3,582 a	20.5 ab	106.5 a	3.25 c–g
RIL_46	33.2 a	2,837 efg	19.7 e	101.5 d	3.16 d–g
RIL_70	21.4 b	2,297 ij	19.8 de	87.6 g	2.85 ghi
RIL_76	27.4 ab	3,287 a–d	20.4 ab	102.5 cd	4.31 a
RIL_89	29.3 a	2,870 d–g	20.1 b–e	101.9 cd	4.03 ab
RIL_102	27.5 ab	2,835 efg	20.3 abc	102.4 cd	3.78 a–d
RIL_103	29.4 a	3,253 a–e	20.3 abc	106.6 a	3.06 efg
RIL_104	28.6 a	3,019 b–g	20.1 b–e	97.2 e	3.88 abc
RIL_113	32.8 a	3,303 abc	20.7 a	105.6 ab	3.53 b-f
RIL_119	33.8 a	2,650 ghi	20.3 abc	107.6 a	3.67 a–e
RIL_122	27.8 ab	3,371 ab	20.6 a	106.2 ab	3.25 c–g
RIL_131	31.2 a	3,290 abc	20.2 bcd	103.3 bcd	2.31 hi
RIL_136	28.6a	3,086 b–f	20.3 abc	105.9 ab	3.09 efg
R99	0^2^	2,400 hij	19.8 de	104.6 abc	2.91 fgh
SEM	2.79	88.8	0.09	0.64	1.77
SEM^3^	2.82			0.66	1.81
	2.84				
	2.85				
	2.90				

1 %Ndfa, nitrogen derived from atmosphere (%); YD, yield (kg ha^−^^1^); δ^13^C, carbon isotope discrimination (‰); DM, maturity (days); HR, harvestability (scale 1 to 5).

2 R99 was used as a reference genotype in calculating %Ndfa and was removed from the analysis of this trait.

3 SEM values were different for treatments with unbalanced data in some traits (highlighted); Tukey–Kramer Grouping for genotype LSmeans (Alpha = 0.05); for each trait, genotype LS-means with the same letter are not significantly different.

Least Squares means (LSmeans) and Standard Error of the Mean (SEM) are presented.

Significant associations were identified among five traits analyzed in 2016 (**Table 4**). Yield was positively correlated with the %Ndfa, δ^13^C, and maturity; δ^13^C was correlated with maturity and harvestability. Significant positive correlation was identified among four nitrogen/rhizobia (C, R, N, and RN) treatments for all analyzed traits (**Supplementary Table S4**).

**Table 4:** Correlations matrix identified among five traits measured in 20 common bean genotypes evaluated at the Elora research station (ON) on N-poor soil in 2016 pilot study using LSmeans data, N = 20.

Traits^1^	YD	δ^13^C	DM	HR
Percent nitrogen derived from atmosphere (%Ndfa, %)	0.451*	0.343	0.124	0.183
Yield (YD, kg ha^−1^)		0.461*	0.537*	0.031
Carbon isotope discrimination (δ^13^C, ‰)			0.593**	0.454*
Maturity (DM, days)				0.196

1 Traits: HR, harvestability (scale 1 to 5).

*significant at the 0.05 level, **significant at the 0.01 level.

#### Experiment 2: Confirmation of Performance of Selected Bean Genotypes in N-Poor Soils

##### Variation in %Ndfa, Yield and Yield-Related Traits

To verify results obtained in 2016, the performances (including nitrogen fixing ability measured as %Ndfa in seed) of 22 bean genotypes [20 genotypes evaluated in 2016 plus OAC Rico (wild type of the non-nodulating mutant, R99) and a black genotype Hi_N_Line] were evaluated at the ERS on low nitrogen soil with four nitrogen/rhizobia treatments in 2017 and 2018. The year was considered a random effect (**Supplementary Table S5**) and only fixed effects in combined analysis were presented (**Table 5**). While the cultivar effect was significant for all traits the effects of nitrogen, rhizobia, and their interactions were not consistent. The nitrogen effect was significant for most of the traits, except δ^13^C and SPAD. In contrast, rhizobia inoculation affected only harvestability. Few interactions were identified including, replace with: R*N for %Ndfa and N*G for harvestability, seed weight and SPAD (**Table 5**).

**Table 5:** Type III tests of fixed effects from restricted likelihood analysis of split-split plot experiments with 22 common bean genotypes conducted at the Elora research station (ON, Canada) on created K-P land in 2017 and 2018 (combined).

Traits^1^	Effect	Numerator DF	Denominator DF	F Value	Pr > F
%Ndfa	Rhizobia (R)	1	3.646	0.87	0.4084
	Nitrogen (N)	1	6.097	460.55	<.0001
	R*N	1	6.097	16.07	0.0068
	Genotype (G)	20	546.3	6.42	<.0001
	R*G	20	546.3	0.84	0.6684
	N*G	20	546.3	0.52	0.9603
	R*N*G	20	546.3	0.58	0.9283
YD	Rhizobia (R)	1	2.715	0.76	0.4525
	Nitrogen (N)	1	6.016	24.30	0.0026
	R*N	1	6.016	0.02	0.8878
	Genotype (G)	21	589.1	20.50	<.0001
	R*G	21	589.1	1.34	0.1400
	N*G	21	589.1	1.54	0.0594
	R*N*G	21	589.1	0.93	0.5487
δ^13^C^NB2^	Rhizobia (R)	1	2.802	7.25	0.0799
	Nitrogen (N)	1	5.992	2.76	0.1476
	R*N	1	5.992	2.31	0.1792
	Genotype (G)	21	587.2	13.89	<.0001
	R*G	21	587.2	1.30	0.1641
	N*G	21	587.2	1.00	0.4628
	R*N*G	21	587.2	1.08	0.3633
DF	Rhizobia (R)	1	2.439	0.15	0.7301
	Nitrogen (N)	1	6.023	14.25	0.0092
	R*N	1	6.023	0.12	0.7444
	Genotype (G)	21	593.9	28.37	<.0001
	R*G	21	593.9	0.82	0.6989
	N*G	21	593.9	0.71	0.8214
	R*N*G	21	593.9	0.26	0.9997
DM^NB^	Rhizobia (R)	1	2.339	0.24	0.6693
	Nitrogen (N)	1	6.019	45.91	0.0005
	R*N	1	6.018	0.48	0.5150
	Genotype (G)	21	592.3	34.18	<.0001
	R*G	21	592.3	1.06	0.3833
	N*G	21	592.3	0.66	0.8713
	R*N*G	21	592.3	0.65	0.8824
PH	Rhizobia (R)	1	4.057	0.00	0.9804
	Nitrogen (N)	1	5.978	60.35	0.0002
	R*N	1	5.978	0.52	0.4963
	Genotype (G)	21	594.9	10.37	<.0001
	R*G	21	594.9	0.66	0.8764
	N*G	21	594.9	0.64	0.8918
	R*N*G	21	594.9	0.99	0.4732
HR^NB^	Rhizobia (R)	1	2.935	11.07	0.0463
	Nitrogen (N)	1	6.01	10.42	0.0179
	R*N	1	6.009	3.75	0.1009
	Genotype (G)	21	596.1	21.23	<.0001
	R*G	21	596.1	0.60	0.9181
	N*G	21	596.1	2.53	0.0002
	R*N*G	21	596.1	1.02	0.4303
SW^NB^	Rhizobia (R)	1	3.069	0.44	0.5552
	Nitrogen (N)	1	5.989	7.02	0.0381
	R*N	1	5.989	0.04	0.8510
	Genotype (G)	21	586.1	74.79	<.0001
	R*G	21	586.1	1.15	0.2893
	N*G	21	586.1	1.96	0.0065
	R*N*G	21	586.1	0.73	0.8004
SPAD^NB^	Rhizobia (R)	1	5.139	1.94	0.2210
	Nitrogen (N)	1	6.027	0.15	0.7135
	R*N	1	6.027	0.00	0.9695
	Genotype (G)	21	593.6	21.69	<.0001
	R*G	21	593.6	0.89	0.5991
	N*G	21	593.6	2.75	<.0001
	R*N*G	21	593.6	0.90	0.5902

1 %Ndfa, percent nitrogen derived from atmosphere (%); YD, yield (kg ha^−1^); δ^13^C, carbon isotope discrimination (‰); DF, flowering (days); DM, maturity (days); PH, plant height (cm); HR, harvestability (scale 1 to 5; SW, seed weight (g); SPAD, leaf chlorophyll content (SPAD units). Non-nodulating genotype R99 was removed from the %Ndfa analysis.

2 Glimmix procedure, combined over two years (2017–2018); Normal distribution, ddfm = kr (NB, nobound option).

The beans under the N treatment had significantly lower values for seed %Ndfa (20.4 *vs.* 45.5%) and SPAD but highest harvestability score (lower value indicates better standability) and seed weight compared to the control (C treatment). They also flowered and matured later; the plants were taller and yielded more (3,004 *vs.* 2,566 kg ha^−1^) when compared to the control. In general, beans under the RN treatment performed similar to the bean plants under the N treatment. They had higher values for %Ndfa (25.4 *vs.* 20.4%) and SPAD but were lower yielding. The addition of rhizobia (R treatment) did not have significant effects on beans. However, there were significant differences in performance between the beans grown under the added mineral nitrogen fertilizer or seed inoculation with rhizobia. The beans under the R treatment had significantly higher values for %Ndfa (42.6 *vs.* 20.4%), matured earlier (97 *vs* 99 days), were shorter (47 *vs.* 50 cm), and had better harvestability (2.1 *vs.* 2.4) but yielded less (2,470 *vs.* 3,004 kg ha^−1^) compared to the beans under the N treatment (**Table 6**).

**Table 6:** Treatment means for nine traits evaluated in 22 common bean genotypes at the Elora research station, ON in 2017 and 2018.

Trait^1^	Treatment^2^				SEM^3^
	C	R	N	RN	
%Ndfa (%)	45.5	42.6	20.4	25.4	1.53/1.54
Yield (kg ha^−1^)	2566	2470	3004	2882	1138.7
δ^13^C (‰)	19.9	19.7	19.7	19.7	0.07
Flowering (days)	47.1	47.3	47.7	47.8	4.92
Maturity (days)	97.0	96.6	99.2	99.2	6.14
Plant height (cm)	46.1	46.5	50.1	49.7	1.13
Harvestability (score 1 to 5)	2.30	2.13	2.36	2.37	0.080/0.081
Seed weight (g)	22.5	22.3	22.9	22.8	0.21
SPAD (SPAD values)	40.1	40.8	40.0	40.7	0.66

1 %Ndfa, percent nitrogen derived from atmosphere (non-nodulating genotype R99 was used as a reference in %Ndfa calculation and was excluded from the trait analysis); δ^13^C, carbon isotope discrimination; SPAD, leaf chlorophyll content.

2 C, control (NoR_NoN); R, rhizobia (R_NoN); N, nitrogen (NoR_N); RN, rhizobia + nitrogen (R_N).

3 SEM values were different for treatments with unbalanced data in some traits (shown underlined). Least Squares means (LSmeans) and Standard Error of the Mean are presented (SEM) (Alpha = 0.05)

There were significant differences among genotypes for %Ndfa, yield, and yield-associated traits, both at the experiment level (**Table 7**) as well as the levels of individual treatments (**Supplementary Table S6**). At the experiment level (all treatments), RIL_119 had the highest %Ndfa value (40.2%) but was late flowering (49 days) and maturing [among the latest (102 days)] and had a yield of 2,785 kg ha^−1^ [which was 17% lower compared to the highest yielding genotype RIL_131 (3,360 kg ha^−1^)] (**Table 7**). However, in the presence of nitrogen fertilizer, its capacity to fix nitrogen was reduced to 48% (control treatment 50.3 *vs.* 26.2% N treatment) but was still among the best nitrogen fixers under the N treatment (after RIL_113 with the %Ndfa value of 26.8%) (**Supplementary Table S6**).

**Table 7:** Performance of 22 common bean genotypes evaluated at the Elora research station, ON in 2017 and 2018.

Genotype	Characteristics evaluated^1^								
	%Ndfa	YD	δ^13^C	DM	HR	DF	PH	SW	SPAD
Mist	28.4 ef	3171 abc	19.5 fgh	97.3 cde	1.28 h	48.2 a	51.1 abc	22.1 g–j	40.9 a–d
Sanilac	29.0 def	2350 gh	20.0 a–e	91.3 g	2.45 b–e	43.7 d	42.9 h	21.2 k	40.9 a–d
RIL_3	28.0 f	2734 def	19.8 c–f	94.7 ef	2.05 efg	45.5 bc	46.8 d–h	23.4 bcd	38.5 ef
RIL_16	37.1 ab	2899 bcd	19.7 e–h	101.9 a	2.27 def	47.8 a	49.1 a–e	22.8 d–g	40.8 a–d
RIL_24	30.9 b–f	2753 def	19.6 e–h	96.2 ed	2.06 ef	45.2 bcd	44.0 gh	23.0 c–f	41.0 a–d
RIL_25	34.8 a–e	2686 d-g	19.7 e–h	97.0 cde	2.19 def	48.2 a	45.4 e–h	22.2 f–i	41.9 abc
TIL_38	37.4 ab	3228 ab	19.9 b–f	100.7 ab	2.20 def	48.3 a	52.6 a	23.1 cde	40.5 a–d
RIL_46	32.1 b–f	2909 bcd	19.4 gh	98.2 bcd	2.09 ef	45.7 b	45.6 e–h	22.5 e–h	42.1 ab
RIL_70	30.0 c–f	2276 h	19.8 c–f	95.4 e	2.83 ab	44.0 cd	44.7 fgh	21.6 ijk	40.7 a-d
RIL_76	31.2 b–f	2458 fgh	19.4 h	95.2 e	2.59 a–d	48.7 a	48.0 b–g	22.8 d–g	42.1 ab
RIL_89	35.1 a–d	2681 d–g	19.6 e–h	96.2 ed	2.89 ab	48.3 a	47.3 c–g	23.7 bc	40.0 cde
RIL_102	33.2 b–f	2639 d–g	20.0 a–d	96.6 ed	2.47 b–e	49.0 a	48.5 a–f	22.8 d–g	42.4 a
RIL_103	32.1 b–f	2981 bcd	20.0 a–e	99.4 abc	2.09 ef	48.6 a	50.0 a–d	23.2 cde	40.7 a–d
RIL_104	31.4 b–f	2505 d–g	20.1 abc	98.2 bcd	2.58 a–d	45.9 b	45.7 e–h	21.9 h–k	40.2 b–e
RIL_113	37.5 ab	2679 d–g	20.2 ab	100.5 ab	2.47 b–e	48.8 a	51.7 ab	23.4 bcd	42.2 a
RIL_119	40.2 a	2785 def	19.7 c–g	101.6 a	2.74 abc	49.0 a	48.6 a–f	24.1 b	40.1 b–e
RIL_122	33.4 b–f	2925 bcd	19.9 a–f	100.9 a	2.20 def	48.8 a	50.3 a–d	21.9 h–k	41.3 a–d
RIL_131	33.1 b–f	3360 a	20.2 a	100.8 ab	1.59 gh	47.5 a	49.3 a–e	25.4 a	39.8 de
RIL_136	35.4 a–d	2838 cde	19.8 c–f	101.6 a	2.11 ef	48.7 a	51.5 ab	21.2 k	40.7 a-d
R99	0^2^	2185 h	19.7 e–h	100.3 ab	1.89 fg	48.4 a	49.2 a–e	19.3 l	34.1 g
OAC_Rico	35.6 abc	2843 cde	19.0 i	99.6 abc	2.30 c–f	48.3 a	49.2 a–e	21.4 jk	40.2 b–e
Hi_N_Line	36.7 ab	2184 h	19.7 d–h	92.1 fg	3.00 a	48.6 a	46.8 d–h	25.1 a	37.2 f
SEM	1.78	1137.4	0.09	6.15	0.113	4.92	1.29	0.20	0.69
SEM^3^	1.77	1137.5			0.114		1.30	0.21	0.70
	1.80	1137.6							
	1.81								
	1.82								
	1.84								
	1.88								

1 %Ndfa, nitrogen derived from atmosphere (%); YD, yield (kg ha^−^^1^); δ^13^C, carbon isotope discrimination (‰); DM, maturity (days); HR, harvestability (scale 1 to 5); DF, flowering (days); PH, plant height (cm); SW, seed weight (g); SPAD, leaf chlorophyll content (SPAD values).

2 R99 was used as a reference genotype in calculating %Ndfa and was removed from the analysis of this trait.

3 SEM values were different for treatments with unbalanced data in some traits (highlighted); Tukey-Kramer Grouping for genotype LSmeans (Alpha=0.05); for each trait, genotype LS-means with the same letter are not significantly different. Least Squares means (LSmeans) and Standard Error of the Mean (SEM) are presented.

The addition of nitrogen fertilizer (N and RN treatments) consistently inhibited the level of nitrogen fixation (calculated as %Ndfa in the seed) in all bean genotypes. However, reduction was variable among the treatments and genotypes. The average reduction was the highest in the N treatment [−55.3% for the genotype average, range −44.2% (RIL_24) to −69.0% (Mist)]. Nitrogen fixation under the RN treatment was reduced by 44.4% [range −29.9% (RIL_119) to −54.6% (RIL_24)]. In general, rhizobia inoculation (R treatment) did not affect %Ndfa, which was similar to that in the control treatment. The differences in the %Ndfa values for the genotype mean between the control and rhizobia inoculated samples was reduced by 6.2% in the 2017–2018 trials. The R induced change ranged from an increase of 8.9% in RIL_70 to a decrease of −20.2% in RIL_46 (**Supplementary Table S6**).

##### Relationships Among the Traits

Few associations were identified between the nine measured traits using both raw data (not shown) and LSmeans in combined 2017–2018 trials. The %Ndfa was significantly positively associated with SPAD and seed weight but was not related to maturity (the possible length of nitrogen fixation cycle). Yield was positively correlated with maturity and plant height and negatively with harvestability. There was also significant positive correlation between flowering and maturity, and both traits were positively associated with plant height. However, the δ^13^C was not significantly associated with any trait analyzed in 2017–2018 (**Table 8**). Significant positive correlations were identified among four nitrogen/rhizobia (C, R, N, and RN) treatments for all analyzed traits (**Supplementary Table S7**).

**Table 8:** Correlations matrix identified among nine traits in 22 common bean genotypes evaluated at the Elora research station (ON) on N-poor soil in 2017 and 2018 using LSmeans data, N = 22.

Traits	YD	δ^13^C	DM	HR	DF	PH	SW	SPAD
%Ndfa	0.398	0.040	0.074	0.346	0.156	0.104	0.624**	0.673**
YD		0.046	0.573**	−0.625**	0.260	0.540**	0.345	0.378
δ^13^C			0.081	0.003	−0.078	0.094	0.254	0.042
DM				−0.346	0.513*	0.706**	−0.074	0.038
HR					−0.038	−0.340	0.184	0.048
DF						0.765**	0.188	−0.037
PH							0.063	−0.016
SW								0.172

1 Traits: %Ndfa, percent nitrogen derived from atmosphere (%); YD, yield (kg ha^−1^); DM, maturity (days); HR, harvestability (scale 1 to 5); DF, flowering (days); PH, plant height (cm); SW, seed weight (g); SPAD, leaf chlorophyll content (SPAD values).

*significant at the 0.05 level, **significant at the 0.01 level.

A principal component analysis (PCA), based on the correlation matrix, was used to reduce the dimensionality of the data. Using the Kaiser criterion (Kaiser, 1958), nine principal components (PC) were identified that had eigenvalues greater than 1 and explained 95.7% [31.11% (PC1) to 1.18% (PC9)] of the variation for traits evaluated at the Elora research station (ON) in 2017–2018, respectively (**Table 9**). However, only the first four PCs explained more than 10% of the variation by themselves.

**Table 9:** **|** Eigenvalues of the correlation matrix from the principal component analysis of the nine traits evaluated in 22 common bean genotypes at the Elora research station, ON in 2017 and 2018.

Order	Eigenvalue	Percentage	Cumulative percentage
1	14.000	31.11	31.11
2	10.352	23.00	54.11
3	6.013	13.36	67.48
4	4.862	10.80	78.28
5	3.834	8.52	86.80
6	1.939	4.31	91.11
7	0.957	2.13	93.24
8	0.582	1.29	94.53
9	0.530	1.18	95.71
10	0.421	0.93	96.64
11	0.314	0.70	97.34
12	0.297	0.66	98.00
13	0.251	0.56	98.56
14	0.157	0.35	98.90
15	0.134	0.30	99.20
16	0.116	0.26	99.46
17	0.076	0.17	99.63
18	0.067	0.15	99.78
19	0.049	0.11	99.89
20	0.035	0.08	99.96
21	0.016	0.04	100.00

**Figure 2:**
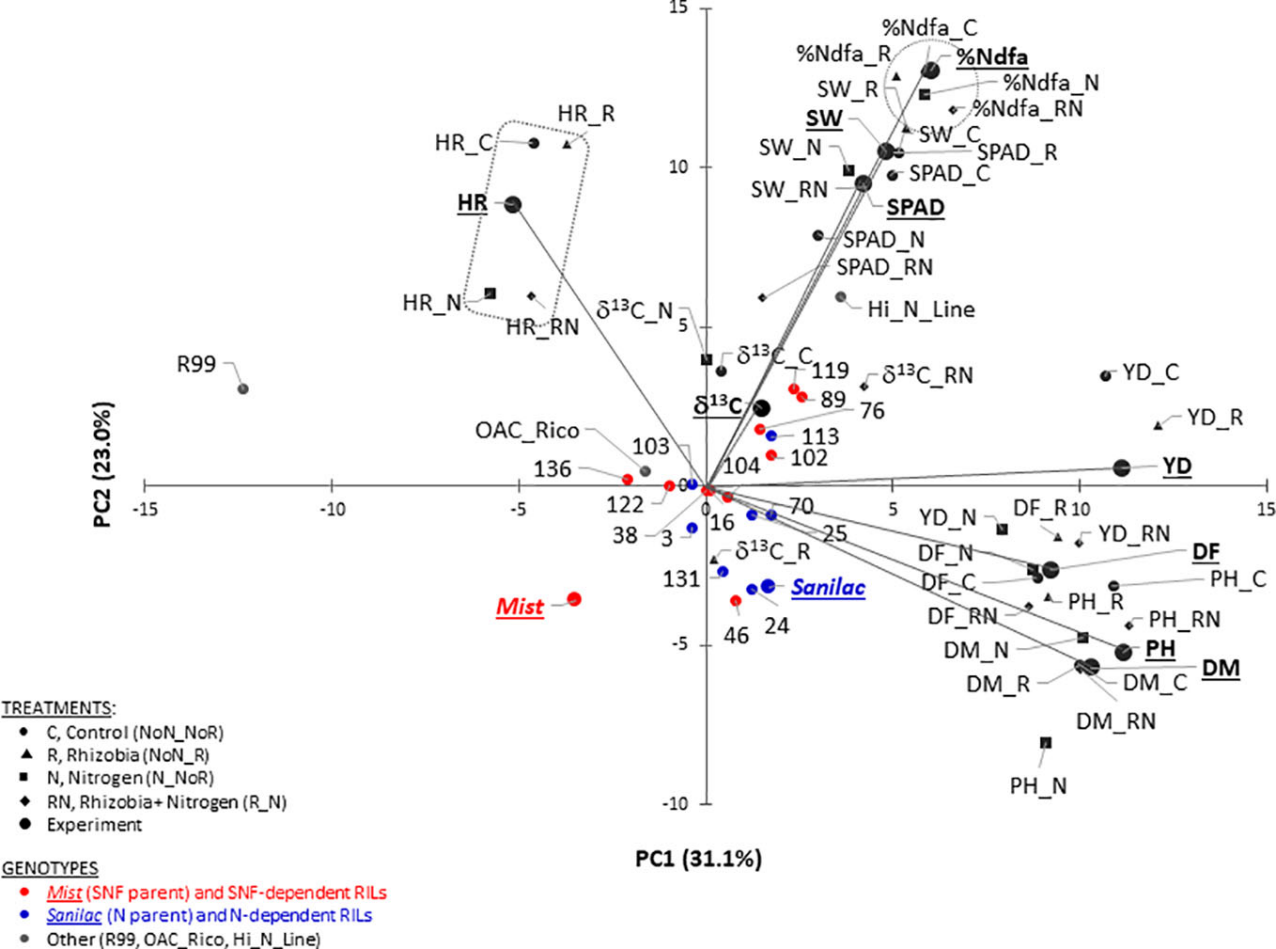
Genotype-trait (GT) biplot analysis of 22 common bean genotypes for traits evaluated at the Elora research station (ERS), ON in 2017 and 2018. Cumulatively, the first two principal components (PC) explained 54.1% of the variability. Traits: percent nitrogen derived from atmosphere (%Ndfa, %), yield (YD, kg ha^-1^), carbon isotope discrimination (δ^13^C, ‰), flowering (DF, days), maturity (DM, days), plant height (PH, cm), harvestability (HR, scale 1 to 5), seed weight (SW, g) and leaf chlorophyll content (SPAD, SPAD values).

Cumulatively, the first two PCs explained 54.1% of the trait’s variability. Averaged over two years, PC1 had large positive associations with yield, flowering, maturity, and plant height. PC2 had large positive associations with seed weight and SPAD (**Figure 2** and **Supplementary Table S8**). Significant associations among traits analyzed under different treatments were indicated by their groupings in a GT biplot. For example, all five harvestability measurements [experiment level and four treatments (C, R, N, and RN)] clustered together in a quadrant opposite to the negatively associated yield trait. However, there is visible separation into a group containing C and R treatments and a group consisting of N and RN treatments, respectively with the experimental value positioned in the middle. Similar clustering was noticeable with five %Ndfa values (**Figure 2**).

Although genotypes were not divided into distinct clusters, the non-nodulating mutant line R99 and black Hi_N_Line were positioned furthest from the rest of the beans. In addition, cultivars Mist (SNF parent, high yield under SNF condition) and Sanilac (N parent, high yield under nitrogen fertilizer) were placed in separate quadrants. Four out of seven N-dependent RILs (RIL_131, RIL_24, RIL_25 and RIL_70) were placed in the same quadrant and close to Sanilac (**Figure 2**).

### Identification of Bean Genotypes That Combine High Yield With Good Nitrogen Fixing Ability (%Ndfa) Over the Three Years

#### Yield and %Ndfa Ratios

**Figure 3:**
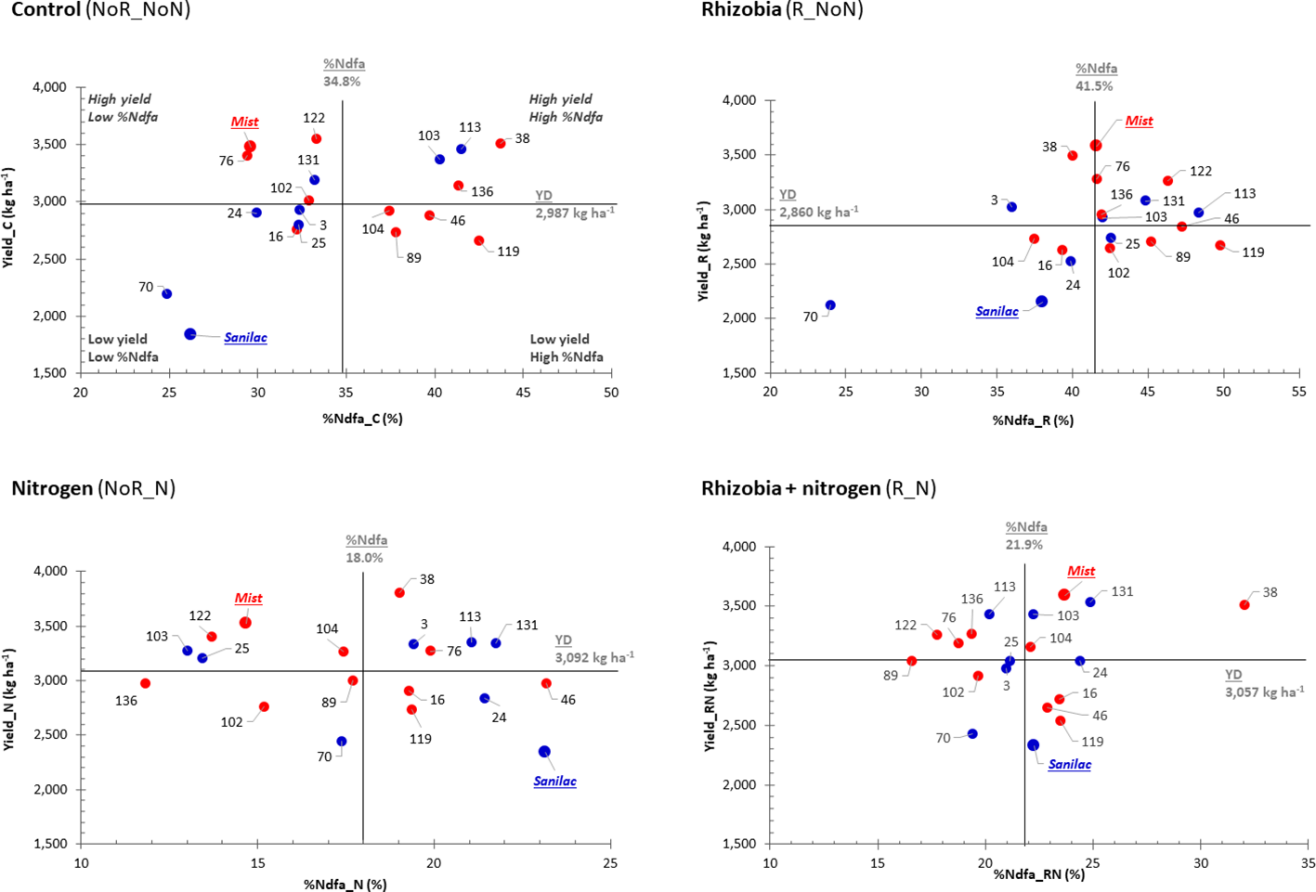
Selection of high-yielding common bean genotypes having a high percentage of nitrogen derived from atmosphere (%Ndfa) in 2016 pilot study. Plots of yield and %Ndfa for 20 bean genotypes in control (C treatment, NoR_NoN), nitrogen (N treatment, NoR_N), rhizobia (R treatment, R_NoN) and combined application of nitrogen fertilizer and rhizobia inoculation (RN treatment, R_N) evaluated at the Elora research station, ON in the 2016 experiment. Plots are divided into four quadrants based on the yield and %Ndfa mean values (black lines): genotypes at the top right quadrant are best performing [they are both high yielding and good nitrogen fixers (%Ndfa)]; genotypes at the top left quadrant are high yielding but low nitrogen fixers; genotypes at the bottom left quadrant are low yielding and low nitrogen fixers; and, genotypes at the bottom right quadrant are low yielding but have good nitrogen fixing abilities. Genotypes: Mist (red, italic), SNF parent (high yield under SNF condition) and 10 SNF-dependent (high yield under SNF condition) RILs (red) and Sanilac (blue, italic), N parent (high yield under nitrogen fertilizer) and seven N-dependent (high yield under nitrogen fertilizer) RILs (blue).

**Figure 4:**
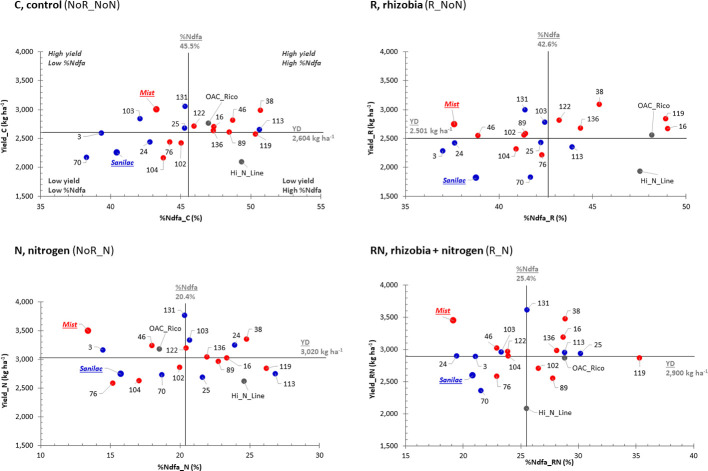
Selection of high-yielding common bean genotypes having a high percentage of nitrogen derived from atmosphere (%Ndfa) in combined 2017-2018 experiments. Yield and %Ndfa plots of 22 bean genotypes in four nitrogen/rhizobia treatments [control (C treatment, NoR_NoN), nitrogen (N treatment, NoR_N), rhizobia (R treatment, R_NoN) and combined application of nitrogen fertilizer and rhizobia inoculation (RN treatment, R_N)] evaluated at the Elora research station, ON in combined 2017_2018 experiment. Plots are divided into four quadrants based on the yield and %Ndfa mean values (black lines): the best performing genotypes are at the top right quadrant [they are both high yielding and good nitrogen fixers )%Ndfa); genotypes at the top left quadrant are high yielding but low nitrogen fixers; genotypes at the bottom left quadrant are low yielding and low nitrogen fixers; and genotypes at the bottom right quadrant are low yielding but have good nitrogen fixing abilities. Genotypes: Mist (red, italic), SNF parent (high yield under SNF condition) and 10 SNF-dependent (high yield under SNF condition) RILs (red) and Sanilac (blue, italic), N parent (high yield under nitrogen fertilizer) and seven N-dependent (high yield under nitrogen fertilizer) RILs (blue).

In 2016, four (C treatments) to seven (R treatment) genotypes displayed high yields with a high nitrogen fixing capacities (%Ndfa) (upper right quadrant, **Figure 3**). Six (R, N, and RN treatments) and eight (C treatment) genotypes were identified in the upper right quadrant having high yield and a high nitrogen fixation (%Ndfa) in 2017–2018 (**Figure 4**). Among them, RIL_38 was the most stable genotype. It had high yields and good %Ndfa capacities in all treatments in both experiments except the R treatment in 2016. Cultivar Mist (upper quadrants) was consistently high yielding but had low nitrogen fixing capacity (except the R and RN treatments in 2016, while Hi_N_Line (bottom right quadrant, analyzed only in 2017 and 2018) had superior nitrogen fixing capacity but had relatively low yields across all treatments. A number of genotypes were identified in the bottom left quadrant having low values for both, yield, and %Ndfa. Among them, RIL_70 was consistently low yielding and inefficient nitrogen fixer across all treatments and experiments. Cultivar Sanilac had also low yields and %Ndfa values in all treatments and years except for the N and RN treatments in 2016 (**Figures 3** and **4**).

#### Genotype Ranks

Classifications based on both experiment level rank (scores 3 and 5, respectively) and treatment sum (Trmt sum = C + R + N + RN) rank (scores 30 and 21, respectively) identified RIL_38 as the best performer in the 2016 pilot study as well as in the 2017–2018 experiments (**Table 10**). This line was also an excellent performer at the level of individual treatments. It ranked first, being both a high yielder and a good nitrogen fixer (%Ndfa), with the addition of nitrogen fertilizer (N treatment), under combined fertilizer and rhizobia inoculation (RN treatment) as well as in the untreated condition (C treatment). When only rhizobia were applied (R treatment), this line ranked second among the 22 genotypes (after RIL_119) in 2017–2018 trials. This line ranked first in 2016 C and RN treatments, third in the N treatment and sixth under the R treatment. RIL_131 also ranked consistently high. It ranked third at the experimental level and was second at the treatment sum level (scores 39 and 48, respectively) in both the 2016 and 2017–2018 trials.

**Table 10:** Ranking of the common bean genotypes based on the yield and %Ndfa values (genotype rank = %Ndfa rank + YD rank).

Genotype rank in 2016 pilot study							2017−2018 genotype rank						
Genotype	Exp^1^	Treatment (Trmt)				Trmt sum	Genotype	Exp	Treatment (Trmt)				Trmt sum
		C	R	N	RN				C	R	N	RN	
RIL_38	**3**	**3**	14	9	**4**	**30**	RIL_38	**5**	**4**	6	**6**	**5**	**21**
RIL_131	10	17	11	**7**	**4**	39	RIL_131	13	11	15	13	9	48
RIL_113	8	7	9	8	16	40	RIL_16	11	15	9	17	8	49
Mist	14	18	12	14	5	49	RIL_119	11	17	**5**	16	16	54
RIL_122	13	10	**8**	16	23	57	RIL_136	16	18	13	18	11	60
RIL_103	13	11	18	22	13	64	RIL_113	17	12	22	16	12	62
RIL_46	16	19	13	13	21	66	RIL_122	15	16	12	18	17	63
RIL_76	18	21	14	13	23	71	RIL_103	16	20	14	14	19	67
RIL_119	18	19	15	24	21	79	OAC_Rico	14	14	14	23	17	68
RIL_136	17	12	18	29	20	79	RIL_46	18	10	28	22	17	77
RIL_3	22	22	23	12	23	80	RIL_25	22	19	24	27	12	82
RIL_104	19	19	28	19	18	84	Mist	21	16	24	23	20	83
RIL_89	19	23	18	20	27	88	RIL_89	22	18	22	19	25	84
RIL_25	23	26	18	24	20	88	RIL_24	26	30	32	10	28	100
RIL_24	23	26	30	19	14	89	RIL_102	27	28	24	26	23	101
RIL_102	25	20	23	28	26	97	Hi_N_Line	27	25	23	25	30	103
RIL_16	26	28	30	22	19	99	RIL_104	30	33	31	37	20	121
Sanilac	32	37	33	21	26	117	RIL_3	31	31	36	30	27	124
RIL_70	33	36	37	29	32	134	RIL_76	32	28	28	41	31	128
R99^2^	33	38	39	31	35	143	RIL_70	36	38	32	31	34	135
Hi_N_Line	NA^3^	NA	NA	NA	NA	NA	Sanilac	36	35	38	34	32	139
OAC_Rico	NA	NA	NA	NA	NA	NA	R99	41	42	42	41	38	163

Genotypes were evaluated at the Elora research station, ON in three years (2016 pilot study and 2017−2018). Within each experiment, genotypes are listed in a descendent order based on the treatment sum (Trmt sum = C + R + N + RN) values.

1 experiment level (all treatments).

2 R99 was used as a non-nodulating reference genotype in %Ndfa calculations and was excluded from the analysis of this trait (%Ndfa for this genotype was set to zero in score calculations); however, this genotype was characterized for all other traits, including yield.

3 not available (not evaluated in 2016).

RIL_16 was third at the treatment sum level in 2017–2018 but was ranked as 15^th^ in 2016. RIL_119 ranked fourth at the treatment sum level in 2017–2018 and was first under the R treatment. This line was ninth in the 2016 trial. Three genotypes (RIL_136, RIL_113 and RIL_122) had similar treatment sum ranks (scores 60 to 63, respectively) in 2017–2018. Among them, RIL_113 was sixth in the 2017–2018 experiment but ranked high third in the 2016 pilot study. RIL_122 was ranked similarly in two experiments, seventh in the 2017–2018 study and fifth in 2016 pilot study. However, this line ranked first in the R treatment in 2016. RIL_136 was more variable; it ranked as fifth in 2017–2018 but was 10^th^ in the 2016 study (**Table 10**).

Interestingly, cultivar Mist [SNF parent (high yield under SNF condition)] ranked 12^th^, in the second half of the 22 genotypes that were evaluated in the 2017–2018 trials of the current study. However, it ranked fourth in the 2016 study, having the second position in the RN treatment and fourth in the R treatment. If R99 was ignored (0 value for %Ndfa, used as a reference in %Ndfa calculation), the cultivar Sanilac [N parent (high yield under nitrogen fertilizer)] and RIL_70 placed at the bottom of the genotype rank list, having low yields and low nitrogen fixing capabilities both, at the experiment level as well as treatment sum level (**Table 10**).

## Discussion

The bean breeding program at the University of Guelph initiated a long term study with the goal of reducing inputs and making bean crops less dependent on nitrogen fertilizers. The results of the current work confirmed the existence of variation in nitrogen fixation (%Ndfa) among the beans from the Sanilac × Mist cross. The genotype effect was significant for all traits, including yield and %Ndfa in both experiments and is consistent with reports in the literature for a strong genetic influence on these traits. For example, Hossain et al. (2017) found significant variation in yield and %Ndfa in a combination of 25 species-cultivar of various pulses (including a single bean cultivar) tested over three years and attributed it both to species/genotypes and environments. Working with beans (selected based on their contrasting nodulation and/or root characteristics), Rodiño et al. (2011) identified significant variation for yield and nodulation in 64 genotypes evaluated (without addition of nitrogen fertilizer or rhizobia inoculants) in six northern Spain environments. Barbosa et al. (2018) identified significant variation for nitrogen fixation associated traits among 100 climbing beans in relation to environmental conditions at two Colombian sites.

### Variability of Nitrogen Fixation Measured as %Ndfa Exist Among Selected Beans

The significant differences identified among 22 genotypes for %Ndfa, yield and yield-related traits, both at the experiment level as well as the levels of individual treatments, suggested that these genotypes may provide a good genetic basis for breeding better nitrogen fixing beans. The general correspondence of the genotypic trends in nitrogen fixing abilities and responses to nitrogen between the previous study by Farid (2015) and the current study also supports this conclusion. However, the relatively low levels of %Ndfa (under 50%) measured for these lines could be attributed to the fact that the majority of the genotypes used in the current study originated from the Mesoamerican (Mist × Sanilac) population of 140 RILs. This population was used previously for mapping a number of nitrogen fixation related traits (Farid, 2015). Although the RILs (17) were selected from the opposite ends of a SNFI index distribution, as SNF-dependent and N-dependent RILs, the genotypes, especially those within the groups, were closely genetically related. Therefore, to capture more variability for the nitrogen fixation related traits, the range of the genotypes should be expanded in future work.

Six traits [yield, %Ndfa, δ^13^C, SPAD (less depending on cumulative effects compared to the others), flowering, and maturity] that were analyzed in this study were in common with the previously reported work that was performed with the whole (140) Mist × Sanilac RIL population (Farid, 2015) but evaluated under different location/nitrogen fertilizer/year conditions. That work also identified a few significant phenotypic correlations among analyzed traits, including flowering positively associated with all traits (maturity r = 0.35*, %Ndfa r = 0.30*, δ^13^C r = 0.16* and SPAD r = 0.17*) except yield, and %Ndfa correlated with maturity (r = 0.19*). No correlation was observed between yield and any of the traits measured. Overall, the associations that were detected in the previous study were different from those we found in the current study, except for a positive association between flowering and maturity in the 2017–2018 experiment (r = 0.51*). Among five traits (yield, maturity, harvestability, %Ndfa, and δ^13^C) that were evaluated in both experiments of the current study, only significant positive correlation between yield and maturity was in common for the two trials. The differences are likely a reflection of the strong effects that environment has in conditioning these traits. The weather conditions, especially rainfall distribution at ERS, were different during the bean growing season in two experiments. Year 2016 received the most rainfall [above the 30-year precipitation average for this site (363 mm)] with the precipitation peaks in July and August. On the other hand, both 2017 and 2018 could be considered as drought-stress environments (Farid and Navabi, 2015) with inadequate precipitation during the periods of seed filling that affected SNF (%Ndfa) and yield.

Some of the correlations observed in the current work have also been identified in other studies. For example, the significant positive association between δ^13^C and maturity identified only in 2016 (r = 0.59**) is in agreement with Wilker et al. (2019) who also identified a significant positive correlation between these two traits in a set of heirloom and conventional cultivars (white and colored beans from both Mesoamerican and Andean gene pools) in two [(r = 0.37* (Elora 2015) and r = 0.48** (Belwood 2015)] out of three Ontario year/location experiments.

Phenotypic correlation among traits is influenced both by genotype and environment. Polania et al. (2016a) identified significant negative correlation of yield with flowering and maturity but positive correlation between yield and seed weight and δ^13^C in experiments with 36 Mesoamerican (bush type) beans in both irrigated and drought trials over two years. Barbosa et al. (2018) identified weak but significant positive correlation between yield and %Ndfa (r = 0.14*) in one location, but that association was not significant in the other Colombian location. Also, these traits were not associated in a set of 42 heirloom and conventional beans analyzed in three location/years experiments (Wilker et al., 2019). Positive associations between %Ndfa and phenological traits (flowering and maturity) that were identified in beans (Farid, 2015; Wilker et al., 2019) may suggest that the selection for improved nitrogen fixation (%Ndfa) can be achieved by extending the flowering time and lengthening the vegetative growth period, thus increasing the availability of photo-assimilates for nodule development (Luthra et al., 1983), but this may result in later maturity. However, in contrast to those studies, the current work did not find an association between %Ndfa and phenological traits. This suggested that flowering and maturity were controlled by fluctuation of precipitation and temperature during bean growing seasons.

Interestingly, a significant positive correlation between %Ndfa and SPAD [measured at mid-pod filling stage (63 DAP)] was identified in the 2017–2018 trials. This correlation may indicate the rate of nitrogen fixation, as suggested in soybean (Vollmann et al., 2011), and agrees with Ramaekers et al. (2013) who suggested that SPAD could be used as a phenotyping tool in breeding for improvement of SNF ability in beans. Significant positive correlations between SPAD (LCS, leaf color score measured six weeks after emergence) and nitrogen fixation (measured as nodule dry weight, NDW) in soybean suggested the use of SPAD as an indicator of SNF effectiveness in soybean (Gwata et al., 2004). In addition, Dinh et al. (2013) identified significant positive correlations among SPAD (75 DAP), pod yield and fixed nitrogen (N-difference method) in *Bradyrhizobium* inoculated peanuts (five genotypes) under two water conditions. Moreover, relatively high heritability for SPAD estimated in maize (Korkovelos and Goulas, 2011) and confirmed in bean (Farid, 2015) suggested the use of SPAD as indirect measure in simultaneous selection for high yield and good ability for nitrogen fixation. However, its efficacy for use as an indirect trait for selecting SNF ability may only be effective if plants are assessed in low nitrogen environments.

The addition of nitrogen fertilizer (100 kg ha^−1^) delayed flowering and maturity, increased yield, but inhibited nitrogen fixation (%Ndfa) in 22 common bean genotypes evaluated in the current study over three years. These are common responses well documented in literature. Numerous studies have shown that nitrogen fixation is inhibited by the addition of nitrogen fertilizer. In soybean, Tamagno et al. (2018) confirmed that nitrogen fertilization reduced SNF but increased yield by enhancing carbon allocation to the seed. However, mechanism is still not clear. Miranda and Bliss (1991) indicated that beans with greater total seed nitrogen were more efficient in partitioning and remobilizing plant nitrogen, rather than or in addition to fixing more nitrogen. However, this inhibition is variable and depends on the applied level of nitrogen fertilizer, genotype and environment (Chidi et al., 2002). On average, the level of %Ndfa was reduced with the addition of 100 kg ha^−1^ nitrogen fertilizer by 47% in 2016 and 55% in 2017–2018. Similar differences in SNF ability and genotypic response were reported by Polania et al. (2016b). They identified an average reduction in SNF ability of 70% in 2012 but 38% in 2013 under drought stress in bush Middle American genotypes using the grain method. In contrast, low levels of nitrogen fertilizer have positive effect on SNF in bean. Argaw and Akuma (2015) reported decrease of nodule number and nodule dry weight with increased rates of nitrogen fertilizer (20, 40 60, 80, and 100 kg ha^−1^) in bean (with or without inoculation) at all experimental sites. In addition, Chekanai et al. (2018) reported that the application of 40 kg ha^−1^ nitrogen significantly increased the number of pods per plant, number of seeds per pod and grain yields. Although the current study utilized the pre-planting nitrogen recommendation for Ontario beans of 100 kg ha^−1^ (OMAFRA, 2017) and a treatment without fertilizer, it would be interesting to test effects of lower levels of nitrogen fertilizer on yield and nitrogen fixation.

Seed inoculation of 22 bean genotypes with rhizobia resulted in minor to no effects on yield and nitrogen fixation (measured as %Ndfa) in the current study. This agreed with other studies reporting that inoculation with rhizobia in field experiments rarely increased yield of beans and gave no significant positive effects in nodulation and biomass production (Hungria and Vargas, 2000; Chekanai et al., 2018). However, there are also reports with successful use of rhizobia inoculation as an alternative nitrogen source in bean production. For example, Beshir et al. (2015) evaluated eight cultivars in factorial combination of three nitrogen treatments (0 and 100 kg N ha^−1^ and *R. etli*) and found that both rhizobia and nitrogen increased total yield of snap beans for 18 and 42%, respectively.

Common bean can be considered as a promiscuous host since a number of rhizobia species are capable of nodulating bean and a diversity of bean-rhizobia interactions exist (Martínez-Roméro, 2003). Positive response to rhizobium inoculation is expected in soils in which the specific rhizobia strains are absent and where indigenous rhizobia are ineffective in nitrogen fixation. However, if abundant native rhizobia strains are present in soils, they would compete with the introduced inoculum to form nodules; moreover, only certain rhizobia strains had the ability to fix nitrogen in specific cultivars (Valverde et al., 2003; Argaw and Muleta, 2017). In this work a commercially available combination of three strains (*Rhizobium leguminosarum* biovar viceae, *Rhizobium leguminosarum* biovar phaseoli and *Bradyrhizobium* sp.) was used. The poor response of common bean to rhizobia inoculation observed in this study could be attributed to failure by the strains that were introduced to adapt to the environmental conditions. However, the soil was not analyzed for the presence and effectiveness of indigenous rhizobia, and any competition among the strains cannot be ruled out.

The effects and interactions observed among bean genotypes, rhizobia strains, nitrogen fertilizer, and environment have been characterized to be of great importance. Gunnabo et al. (2019) evaluated several rhizobia strains with a variety of bean genotypes (selected from a set of 192 Mesoamerican and Andean beans) in East African (Ethiopia and Kenya) environments. The rhizobia strain CIAT899 was superior, but significant genotype × rhizobia strain was identified implying “either opportunities or challenges for enhancing nitrogen fixation”. In the current study, the genotype effect was significant for all traits in all three years, while the effects of nitrogen, rhizobia, and their interactions were inconsistent among experiments. Significant interactions indicated that the seed inoculation with rhizobia and addition of nitrogen fertilizer affected nitrogen fixation (%Ndfa), yield, and yield-related traits in 22 genotypes in different ways (Rennie and Kemp, 1983a; Rennie and Kemp, 1983b).

In general, selection of high yielding genotypes with good ability for nitrogen fixation (%Ndfa) has been slow in beans. Selection for this trait is hampered by low and/or inconsistent correlations between the yield and %Ndfa, as reported previously (Farid, 2015; Polania et al., 2016b) and confirmed in the current study. Close associations among %Ndfa, SPAD, and seed weight indicated that a correlated response to selection can be expected with selection for any of these traits. However, the lack of, or inconsistent association between %Ndfa and yield suggests that selection for high nitrogen fixing (%Ndfa) genotypes would not necessarily result in greater yields and that selection for both traits should be performed simultaneously. Indices to quantify nitrogen use efficiency that can be calculated from the measured yield and nitrogen fixation (%Ndfa) data could be useful in ranking genotypes, especially for sensitivity to the application of nitrogen fertilizers. They could be used to select for multiple traits, including high yield and sensitivity to nitrogen fertilizers. It is interesting to examine how successful such indices might be with the genetic material used in the current study. Using two yield-based indices [Nitrogen Stability Index (NSI), Smith et al., 2012; SNF relative efficiency index (SNFI), Farid, 2015], developed previously by our group, all 22 genotypes were in general nitrogen-responsive and were not significantly different (data not shown).

### High Yielding Beans With Good %Ndfa Were Identified

The sensitivity of legume–rhizobia relationships to nitrogen fertilizers varies among species (Westermann et al., 1981) and, as was confirmed in the current study, among beans. Genotypes and cultivars with enhanced nodulation and nitrogen fixation capacity have been identified in a number of studies (Pereira and Bliss, 1989; Akter et al., 2017); however, many of the high nitrogen fixing bean lines were selected in the absence or at low rates of nitrate (Rennie and Kemp, 1983b). Park and Buttery (1989) identified genotypes with superior nodulation and nitrogen fixation capacity in the presence of nitrogen in replicated pot trials. They suggested that these genotypes would be good candidates for improving the nitrate tolerant nodulating characteristic of bean. Recently, Akter et al. (2017) identified several bean genotypes with good fixing ability in Alberta (Western Canada) environments and suggested that the efficient nitrogen-responsive cultivars may enable common bean growers to reduce nitrogen input without compromising yield and seed quality.

The current study identified a few high yielding genotypes with good nitrogen fixing capabilities (measured as %Ndfa). Genotypes like RIL_38 (high for both yield and %Ndfa, responsive to both low and high nitrogen), Mist (consistently high yielding), and Hi_N_Line (high %Ndfa black bean) were identified in this study as good candidates for breeding high yielding and/or superior nitrogen fixers. Classification of the bean genotypes based on all treatment rank (C + R + N + RN) for the combined yield and %Ndfa values identified RIL_38 and RIL_131 as the best performers, both in the 2016 pilot study as well as in the 2017−2018 combined experiment. RIL_38 was the best in combining high yield with greater %Ndfa in all treatments in both experiments. A few additional lines also ranked consistently high. RIL_113 ranked as the third in 2016 and sixth in 2017–2018 while RIL_119 ranked as the fourth in 2017–2018 and ninth in 2016.

High yield is still the major objective in most bean breading programs. Under the fertilized conditions, yield increase of 8.4% in 2016 (3,807 kg ha^−1^ in the N treatment and 3,511 kg ha^−1^ in the non-fertilized C treatment) and 12% in 2017–2018 (3,346 kg ha^−1^ in the N treatment and 2,988 kg ha^−1^ in the C treatment), respectively was identified in RIL_38. At the same time, %Ndfa reduction of 56.5% (43.7% in the C treatment and 19.0% in the N treatment) in 2016 and 51.1% (50.7% in the C treatment and 24.8% in the N treatment) in 2017–2018, respectively was observed. Similar response was observed in the second ranking line, RIL_131 suggesting that nitrogen fixation (%Ndfa) in these genotypes was less inhibited by the addition of nitrogen fertilizer.

## Conclusions

The study confirmed the strong negative effect that nitrogen fertilizer has on the capacity of common bean to utilize atmospheric nitrogen through its symbiotic association with rhizobium and emphasizes the benefit of research to improve the nitrogen fixing capacity of this crop. The ineffectiveness of inoculations with rhizobia and the importance of environmental factors affecting SNF were also observed. The addition of nitrogen delayed flowering, maturity, and increased plant height and yield, while the application of rhizobia had significant effects only on few traits. Nitrogen application reduced SNF (%Ndfa), but the effect was variable among genotypes and years, confirming importance of both genotype and environment in expression of these traits.

This work identified genotypes that can be used in the selection of beans with less sensitivity to nitrogen fertilizer. Four genotypes can be grown either in low or high nitrogen environments. Among them, line RIL_38 was the most stable, showing consistently high yields and good nitrogen fixation capacities (%Ndfa). In some years, a few genotypes performed better in low nitrogen soils than in high nitrogen soil. However, their performance was inconsistent. The cultivar Mist and a Hi_N_Line were identified as good genotypes for breeding high yielding or superior nitrogen fixing cultivars, respectively. Collectively, the study identified genotypes that have the potential for reducing inputs and making bean crops less dependent on nitrogen fertilizers.

Additional studies with these lines may lead to an understanding of the phenomenon of nitrogen fixation suppression by nitrogen fertilizer. In addition, lines like RIL_38 would be the most suitable genotype to use in a reduced input production system due to its stable high nitrogen fixation (%Ndfa) and yield with and without nitrogen and/or rhizobia. However, the suitability of these lines for breeding high yielding, nitrate insensitive, efficient nitrogen fixing bean cultivars, needs to be confirmed by additional testing with different rhizobia strains and nitrogen treatments, including low nitrogen soil levels and with a knowledge of the indigenous rhizobia in the soils. The results of the current work indicate that selection under low and/or high soil nitrogen environments may result in the development of high yielding lines with good nitrogen fixing capabilities.

## Data Availability Statement

The datasets generated for this study are available on request to the corresponding author.

## Author Contributions

KPP designed the project. KPP, THS, and YR conceived and planned the work. THS and LS set up experiments and performed field work. Crop data were collected by LS and YR. FM and BH were responsible for the IRMS seed analysis. YR analyzed the data and wrote first draft of the manuscript. KPP revised and proofread the manuscript.

## Funding

This project is a part of the Food From Thought research program at the University of Guelph. The research was undertaken in part thanks to generous funding from the Canada First Research Excellence Fund. Current study was also funded by the Ontario Bean Growers, Agriculture and Agri-Food Canada, the Ontario Ministry for Food and Rural Affairs and the Ontario Ministry for Research and Innovation. 

## Conflict of Interest

The authors declare that the research was conducted in the absence of any commercial or financial relationships that could be construed as a potential conflict of interest.
