# Supplementary material for: Effects of Nitrogen Application on Nitrogen Fixation in Common Bean Production
Source: Front Plant Sci. 2020 Aug 6;11:1172. doi: 10.3389/fpls.2020.01172 (PMC7424037; doi:10.3389/fpls.2020.01172)
Supplement: Supplementary file 1 [file Presentation_1.pptx]

## Slide 1
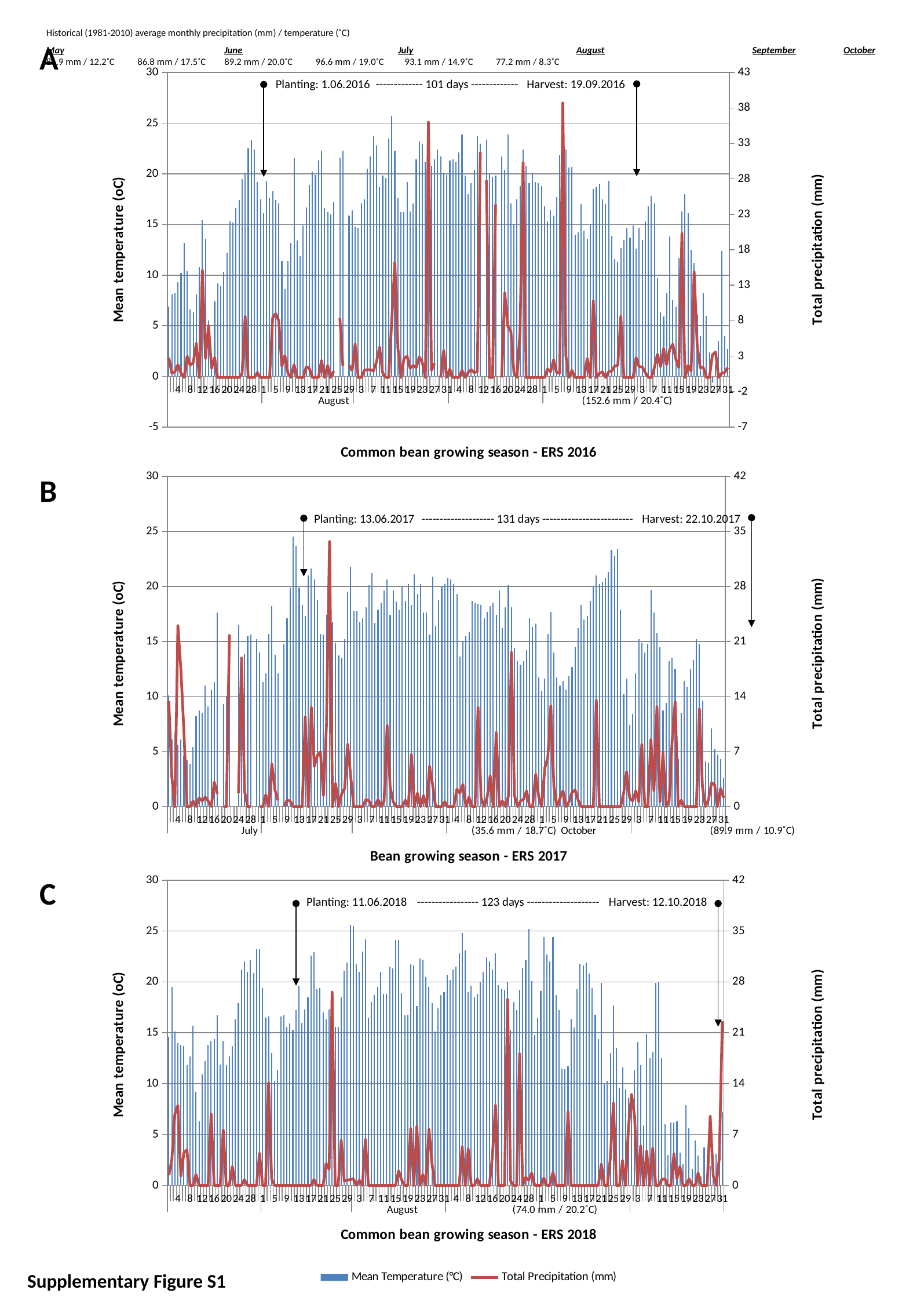

Historical (1981-2010) average monthly precipitation (mm) / temperature (˚C)
May		 June		 July		 August		 September	 October
86.9 mm / 12.2˚C	 86.8 mm / 17.5˚C	 89.2 mm / 20.0˚C	 96.6 mm / 19.0˚C	 93.1 mm / 14.9˚C	 77.2 mm / 8.3˚C
A
### Chart
| Category | Mean Temperature (°C) | Total Precipitation (mm) |
|---|---|---|
| 1 | 6.9 | 2.8 |
| 2 | 8.1 | 0.6000000000000006 |
| 3 | 8.200000000000001 | 0.8 |
| 4 | 9.3 | 1.8 |
| 5 | 10.200000000000001 | 0.6000000000000006 |
| 6 | 13.2 | 0.0 |
| 7 | 10.4 | 3.0 |
| 8 | 6.6 | 1.7000000000000004 |
| 9 | 6.3 | 2.1 |
| 10 | 8.1 | 4.8 |
| 11 | 10.8 | 0.0 |
| 12 | 15.4 | 15.1 |
| 13 | 13.6 | 2.7 |
| 14 | 5.5 | 7.3 |
| 15 | 2.1 | 1.3 |
| 16 | 7.4 | 2.8 |
| 17 | 9.200000000000001 | 0.0 |
| 18 | 8.9 | 0.0 |
| 19 | 10.3 | 0.0 |
| 20 | 12.2 | 0.0 |
| 21 | 15.3 | 0.0 |
| 22 | 15.2 | 0.0 |
| 23 | 16.6 | 0.0 |
| 24 | 17.4 | 0.0 |
| 25 | 19.5 | 0.6000000000000006 |
| 26 | 20.1 | 8.6 |
| 27 | 22.5 | 0.0 |
| 28 | 23.3 | 0.0 |
| 29 | 22.4 | 0.0 |
| 30 | 19.2 | 0.7000000000000006 |
| 31 | 17.5 | 0.0 |
| 1 | 16.1 | 0.0 |
| 2 | 19.3 | 0.0 |
| 3 | 17.6 | 0.0 |
| 4 | 18.3 | 8.4 |
| 5 | 17.4 | 9.0 |
| 6 | 17.1 | 8.0 |
| 7 | 11.4 | 1.6 |
| 8 | 8.6 | 3.1 |
| 9 | 11.4 | 0.6000000000000006 |
| 10 | 13.2 | 0.0 |
| 11 | 21.6 | 1.8 |
| 12 | 13.4 | 0.0 |
| 13 | 11.9 | 0.0 |
| 14 | 14.9 | 0.0 |
| 15 | 16.7 | 1.5 |
| 16 | 18.9 | 1.3 |
| 17 | 20.2 | 0.0 |
| 18 | 19.9 | 0.0 |
| 19 | 21.3 | 0.0 |
| 20 | 22.3 | 2.4 |
| 21 | 16.6 | 0.0 |
| 22 | 16.2 | 1.7000000000000004 |
| 23 | 16.0 | 0.0 |
| 24 | 17.2 | 0.9 |
| 25 | None | None |
| 26 | 21.6 | 8.4 |
| 27 | 22.3 | 1.6 |
| 28 | None | None |
| 29 | 15.9 | 1.7000000000000004 |
| 30 | 16.4 | 1.0 |
| 1 | 14.8 | 4.7 |
| 2 | 14.7 | 0.0 |
| 3 | 17.1 | 0.0 |
| 4 | 17.5 | 1.0 |
| 5 | 20.5 | 1.1 |
| 6 | 21.7 | 1.1 |
| 7 | 23.7 | 0.9 |
| 8 | 22.8 | 2.2 |
| 9 | 18.7 | 4.3 |
| 10 | 19.8 | 0.7000000000000006 |
| 11 | 19.6 | 0.0 |
| 12 | 23.5 | 0.0 |
| 13 | 25.7 | 8.1 |
| 14 | 22.3 | 16.2 |
| 15 | 17.6 | 4.2 |
| 16 | 16.2 | 0.0 |
| 17 | 16.2 | 2.7 |
| 18 | 19.2 | 3.0 |
| 19 | 16.3 | 1.3 |
| 20 | 17.1 | 1.7000000000000004 |
| 21 | 21.4 | 1.4 |
| 22 | 23.2 | 2.9 |
| 23 | 23.0 | 2.1 |
| 24 | 21.2 | 0.0 |
| 25 | 22.4 | 36.0 |
| 26 | 20.8 | 1.0 |
| 27 | 21.4 | 2.0 |
| 28 | 22.4 | None |
| 29 | 21.7 | 0.0 |
| 30 | 20.1 | 3.8 |
| 31 | 19.9 | 0.0 |
| 1 | 21.3 | 1.2 |
| 2 | 21.4 | 0.0 |
| 3 | 21.2 | 0.0 |
| 4 | 22.1 | 0.0 |
| 5 | 23.9 | 1.0 |
| 6 | 19.8 | 0.0 |
| 7 | 18.0 | 0.7000000000000006 |
| 8 | 19.1 | 1.1 |
| 9 | 20.4 | 0.7000000000000006 |
| 10 | 23.7 | 0.8 |
| 11 | 23.0 | 31.8 |
| 12 | None | None |
| 13 | 23.4 | 27.8 |
| 14 | 20.0 | 0.0 |
| 15 | 19.7 | 0.0 |
| 16 | 19.8 | 24.4 |
| 17 | None | None |
| 18 | 21.7 | 0.0 |
| 19 | 20.4 | 11.9 |
| 20 | 23.9 | 7.2 |
| 21 | 17.1 | 6.5 |
| 22 | 15.0 | 0.7000000000000006 |
| 23 | 17.5 | 0.0 |
| 24 | 18.8 | 6.5 |
| 25 | 22.4 | 30.3 |
| 26 | 20.8 | 0.0 |
| 27 | 19.1 | 0.0 |
| 28 | 20.1 | 0.0 |
| 29 | 19.2 | 0.0 |
| 30 | 19.1 | 0.0 |
| 31 | 18.8 | 0.0 |
| 1 | 16.8 | 0.0 |
| 2 | 15.3 | 1.2 |
| 3 | 16.4 | 0.8 |
| 4 | 15.9 | 2.5 |
| 5 | 17.7 | 0.7000000000000006 |
| 6 | 21.8 | 0.6000000000000006 |
| 7 | 24.3 | 38.7 |
| 8 | 22.4 | 3.1 |
| 9 | 20.6 | 0.0 |
| 10 | 20.7 | 1.0 |
| 11 | 14.0 | 0.0 |
| 12 | 14.2 | 0.0 |
| 13 | 17.0 | 0.0 |
| 14 | 14.4 | 0.0 |
| 15 | 13.6 | 2.7 |
| 16 | 15.0 | 0.0 |
| 17 | 18.5 | 10.8 |
| 18 | 18.7 | 0.0 |
| 19 | 19.0 | 0.6000000000000006 |
| 20 | 17.5 | 0.8 |
| 21 | 17.0 | 0.0 |
| 22 | 19.3 | 0.8 |
| 23 | 13.9 | 0.9 |
| 24 | 11.6 | 1.6 |
| 25 | 11.3 | 1.7000000000000004 |
| 26 | 12.7 | 8.6 |
| 27 | 13.5 | 0.0 |
| 28 | 14.6 | 0.0 |
| 29 | 13.7 | 0.0 |
| 30 | 14.9 | 0.0 |
| 1 | 12.6 | 2.8 |
| 2 | 14.7 | 1.5 |
| 3 | 13.5 | 1.5 |
| 4 | 15.3 | 0.7000000000000006 |
| 5 | 16.8 | 0.0 |
| 6 | 17.8 | 0.0 |
| 7 | 17.1 | 1.4 |
| 8 | 9.700000000000001 | 3.3 |
| 9 | 6.3 | 1.5 |
| 10 | 5.9 | 4.1 |
| 11 | 8.200000000000001 | 1.8 |
| 12 | 13.8 | 3.9 |
| 13 | 7.5 | 4.7 |
| 14 | 6.9 | 2.6 |
| 15 | 11.7 | 1.4 |
| 16 | 16.3 | 20.3 |
| 17 | 18.0 | 0.0 |
| 18 | 16.1 | 1.7000000000000004 |
| 19 | 12.5 | 0.9 |
| 20 | 11.2 | 14.9 |
| 21 | 6.1 | 4.5 |
| 22 | 4.0 | 1.4 |
| 23 | 8.200000000000001 | 1.4 |
| 24 | 6.0 | 0.0 |
| 25 | 2.4 | 0.0 |
| 26 | -0.6000000000000006 | 3.2 |
| 27 | 1.2 | 3.6 |
| 28 | 3.5 | 0.0 |
| 29 | 12.4 | 0.6000000000000006 |
| 30 | 4.0 | 0.7000000000000006 |
| 31 | 2.7 | 1.4 |Planting: 1.06.2016
------------- 101 days -------------
Harvest: 19.09.2016
### Chart
| Category | Mean Temperature (°C) | Total Precipitation (mm) |
|---|---|---|
| 1 | 10.1 | 13.4 |
| 2 | 6.1 | 3.8 |
| 3 | 6.7 | 0.0 |
| 4 | 5.6 | 23.0 |
| 5 | 6.1 | 17.4 |
| 6 | 4.6 | 9.4 |
| 7 | 4.2 | 0.0 |
| 8 | 3.9 | 0.0 |
| 9 | 5.4 | 0.7000000000000006 |
| 10 | 8.200000000000001 | 0.0 |
| 11 | 8.700000000000001 | 1.1 |
| 12 | 8.5 | 0.7000000000000006 |
| 13 | 11.0 | 1.2 |
| 14 | 9.1 | 0.7000000000000006 |
| 15 | 10.6 | 0.0 |
| 16 | 11.3 | 3.1 |
| 17 | 17.6 | 1.6 |
| 18 | None | None |
| 19 | 9.3 | 0.0 |
| 20 | 10.0 | 0.0 |
| 21 | 11.6 | 21.9 |
| 22 | None | None |
| 23 | None | None |
| 24 | 16.5 | 1.7 |
| 25 | 11.9 | 18.9 |
| 26 | 13.9 | 1.9000000000000001 |
| 27 | 15.5 | 0.0 |
| 28 | 15.6 | 0.0 |
| 29 | None | None |
| 30 | 15.2 | None |
| 31 | 14.0 | 0.0 |
| 1 | 11.3 | 0.0 |
| 2 | 12.1 | 1.5 |
| 3 | 15.7 | 0.0 |
| 4 | 18.2 | 5.4 |
| 5 | 13.8 | 2.1 |
| 6 | 12.1 | 0.8 |
| 7 | None | None |
| 8 | 14.8 | 0.0 |
| 9 | 17.1 | 0.8 |
| 10 | 19.9 | 0.7000000000000006 |
| 11 | 24.5 | 0.0 |
| 12 | 23.7 | 0.0 |
| 13 | 19.9 | 0.0 |
| 14 | 18.3 | 0.0 |
| 15 | 17.3 | 11.4 |
| 16 | 21.0 | 0.0 |
| 17 | 21.6 | 12.6 |
| 18 | 20.6 | 5.1 |
| 19 | 18.8 | 6.5 |
| 20 | 15.7 | 6.9 |
| 21 | 15.6 | 1.4 |
| 22 | 17.4 | 10.4 |
| 23 | 19.0 | 33.7 |
| 24 | 16.8 | 0.0 |
| 25 | 14.9 | 2.9 |
| 26 | 13.7 | 0.0 |
| 27 | 13.5 | 1.6 |
| 28 | 15.2 | 2.3 |
| 29 | 19.5 | 7.9 |
| 30 | 21.8 | 3.8 |
| 1 | 17.8 | 0.0 |
| 2 | 17.8 | 0.0 |
| 3 | 16.8 | 0.0 |
| 4 | 17.1 | 0.0 |
| 5 | 18.1 | 0.9 |
| 6 | 20.1 | 0.7000000000000006 |
| 7 | 21.2 | 0.0 |
| 8 | 16.7 | 0.0 |
| 9 | 17.9 | 0.9 |
| 10 | 18.5 | 0.0 |
| 11 | 19.6 | 0.7000000000000006 |
| 12 | 20.6 | 10.3 |
| 13 | 17.4 | 2.5 |
| 14 | 19.6 | 0.6000000000000006 |
| 15 | 18.6 | 0.0 |
| 16 | 17.9 | 0.0 |
| 17 | 20.0 | 0.0 |
| 18 | 18.7 | 0.8 |
| 19 | 20.2 | 0.0 |
| 20 | 18.3 | 6.6 |
| 21 | 21.1 | 0.0 |
| 22 | 19.3 | 1.7 |
| 23 | 20.2 | 0.0 |
| 24 | 17.6 | 1.4 |
| 25 | 17.6 | 0.0 |
| 26 | 15.6 | 5.1 |
| 27 | 20.9 | 2.8 |
| 28 | 16.4 | 0.0 |
| 29 | 18.8 | 0.0 |
| 30 | 20.0 | 0.0 |
| 31 | 20.2 | 0.6000000000000006 |
| 1 | 20.8 | 0.0 |
| 2 | 20.6 | 0.0 |
| 3 | 20.2 | 0.0 |
| 4 | 19.3 | 2.2 |
| 5 | 13.6 | 1.7 |
| 6 | 15.0 | 2.8 |
| 7 | 15.5 | 0.0 |
| 8 | 15.9 | 1.2 |
| 9 | 18.7 | 0.0 |
| 10 | 18.5 | 0.0 |
| 11 | 18.4 | 12.6 |
| 12 | 18.3 | 1.1 |
| 13 | 17.1 | 0.0 |
| 14 | 17.7 | 1.2 |
| 15 | 18.2 | 3.9 |
| 16 | 18.5 | 0.0 |
| 17 | 17.4 | 9.4 |
| 18 | 19.6 | 0.0 |
| 19 | 16.2 | 0.7000000000000006 |
| 20 | 18.1 | 0.0 |
| 21 | 20.1 | 1.3 |
| 22 | 18.1 | 19.6 |
| 23 | 14.4 | 1.4 |
| 24 | 13.2 | 0.0 |
| 25 | 12.9 | 0.7000000000000006 |
| 26 | 13.2 | 0.9 |
| 27 | 14.2 | 2.0 |
| 28 | 17.1 | 0.0 |
| 29 | 16.3 | 0.0 |
| 30 | 16.6 | 4.1 |
| 31 | 11.7 | 1.3 |
| 1 | 10.5 | 0.0 |
| 2 | 11.6 | 4.9 |
| 3 | 15.7 | 6.2 |
| 4 | 17.7 | 12.8 |
| 5 | 14.0 | 2.6 |
| 6 | 11.7 | 0.0 |
| 7 | 11.0 | 1.0 |
| 8 | 11.4 | 2.0 |
| 9 | 10.6 | 0.0 |
| 10 | 11.9 | 0.6000000000000006 |
| 11 | 12.7 | 1.8 |
| 12 | 14.5 | 2.1 |
| 13 | 16.2 | 0.8 |
| 14 | 18.3 | 0.0 |
| 15 | 17.0 | 0.0 |
| 16 | 17.3 | 0.0 |
| 17 | 18.7 | 0.0 |
| 18 | 20.0 | 0.0 |
| 19 | 21.0 | 13.5 |
| 20 | 20.2 | 0.0 |
| 21 | 20.4 | 0.0 |
| 22 | 20.8 | 0.0 |
| 23 | 21.3 | 0.0 |
| 24 | 23.3 | 0.0 |
| 25 | 22.8 | 0.0 |
| 26 | 23.4 | 0.0 |
| 27 | 17.9 | 0.0 |
| 28 | 10.200000000000001 | 1.7 |
| 29 | 11.6 | 4.4 |
| 30 | 7.4 | 1.1 |
| 1 | 8.4 | 0.7000000000000006 |
| 2 | 12.1 | 2.0 |
| 3 | 15.2 | 0.6000000000000006 |
| 4 | 14.9 | 7.9 |
| 5 | 14.0 | 0.0 |
| 6 | 14.8 | 0.0 |
| 7 | 19.7 | 8.5 |
| 8 | 17.6 | 2.0 |
| 9 | 15.8 | 12.7 |
| 10 | 14.5 | 0.6000000000000006 |
| 11 | 8.700000000000001 | 6.8 |
| 12 | 9.4 | 0.0 |
| 13 | 13.2 | 0.7000000000000006 |
| 14 | 13.5 | 8.3 |
| 15 | 12.5 | 13.3 |
| 16 | 4.3 | 0.0 |
| 17 | 8.5 | 0.8 |
| 18 | 11.4 | 0.0 |
| 19 | 10.9 | 0.0 |
| 20 | 12.5 | 0.0 |
| 21 | 13.3 | 0.0 |
| 22 | 15.2 | 0.0 |
| 23 | 14.8 | 12.4 |
| 24 | 9.6 | 2.5 |
| 25 | 4.1 | 0.0 |
| 26 | 4.0 | 0.9 |
| 27 | 7.1 | 3.0 |
| 28 | 5.2 | 2.8 |
| 29 | 4.7 | 0.0 |
| 30 | 4.3 | 2.3 |
| 31 | 2.6 | 1.1 |B
Planting: 13.06.2017
Harvest: 22.10.2017
-------------------- 131 days -------------------------
### Chart
| Category | Mean Temperature (°C) | Total Precipitation (mm) |
|---|---|---|
| 1 | 14.6 | 1.4 |
| 2 | 19.5 | 3.5 |
| 3 | 15.1 | 9.8 |
| 4 | 14.0 | 11.0 |
| 5 | 13.8 | 1.3 |
| 6 | 13.7 | 4.5 |
| 7 | 11.8 | 4.9 |
| 8 | 12.7 | 0.0 |
| 9 | 15.7 | 0.0 |
| 10 | 9.200000000000001 | 1.5 |
| 11 | 6.3 | 0.0 |
| 12 | 10.9 | 0.0 |
| 13 | 12.2 | 0.0 |
| 14 | 13.8 | 0.0 |
| 15 | 14.2 | 9.8 |
| 16 | 14.4 | 0.0 |
| 17 | 16.7 | 0.0 |
| 18 | 11.9 | 0.0 |
| 19 | 14.2 | 7.6 |
| 20 | 11.8 | 0.0 |
| 21 | 12.7 | 0.0 |
| 22 | 13.7 | 2.6 |
| 23 | 16.3 | 0.0 |
| 24 | 17.9 | 0.0 |
| 25 | 21.2 | 0.0 |
| 26 | 22.0 | 0.8 |
| 27 | 21.0 | 0.0 |
| 28 | 22.1 | 0.0 |
| 29 | 20.9 | 0.0 |
| 30 | 23.2 | 0.0 |
| 31 | 23.2 | 4.4 |
| 1 | 19.4 | 0.0 |
| 2 | 16.5 | 0.0 |
| 3 | 16.6 | 14.1 |
| 4 | 13.0 | 0.9 |
| 5 | 10.200000000000001 | 0.0 |
| 6 | 11.3 | 0.0 |
| 7 | 16.6 | 0.0 |
| 8 | 16.7 | 0.0 |
| 9 | 15.6 | 0.0 |
| 10 | 15.9 | 0.0 |
| 11 | 15.3 | 0.0 |
| 12 | 17.2 | 0.0 |
| 13 | 19.6 | 0.0 |
| 14 | 16.0 | 0.0 |
| 15 | 17.3 | 0.0 |
| 16 | 18.5 | 0.0 |
| 17 | 22.6 | 0.0 |
| 18 | 22.9 | 0.8 |
| 19 | 19.3 | 0.0 |
| 20 | 19.4 | 0.0 |
| 21 | 17.0 | 0.0 |
| 22 | 16.3 | 3.0 |
| 23 | 17.3 | 2.2 |
| 24 | 16.4 | 26.6 |
| 25 | 15.6 | 0.0 |
| 26 | 15.6 | 0.0 |
| 27 | 18.5 | 6.2 |
| 28 | 21.1 | 0.6000000000000006 |
| 29 | 21.9 | 0.7000000000000006 |
| 30 | 25.6 | 0.8 |
| 1 | 25.5 | 0.9 |
| 2 | 21.7 | 0.0 |
| 3 | 21.0 | 0.7000000000000006 |
| 4 | 23.0 | 0.0 |
| 5 | 24.2 | 6.3 |
| 6 | 16.5 | 0.0 |
| 7 | 18.0 | 0.0 |
| 8 | 18.7 | 0.0 |
| 9 | 19.5 | 0.0 |
| 10 | 21.0 | 0.0 |
| 11 | 18.8 | 0.0 |
| 12 | 18.8 | 0.0 |
| 13 | 21.5 | 0.0 |
| 14 | 21.3 | 0.0 |
| 15 | 24.1 | 0.0 |
| 16 | 24.1 | 2.0 |
| 17 | 18.9 | 0.7000000000000006 |
| 18 | 16.7 | 0.0 |
| 19 | 16.8 | 0.0 |
| 20 | 21.7 | 7.8 |
| 21 | 21.6 | 0.0 |
| 22 | 17.6 | 8.1 |
| 23 | 22.3 | 0.0 |
| 24 | 22.2 | 1.5 |
| 25 | 20.5 | 0.0 |
| 26 | 19.5 | 7.7 |
| 27 | 17.9 | 3.0 |
| 28 | 15.1 | 0.0 |
| 29 | 17.4 | 0.0 |
| 30 | 18.7 | 0.0 |
| 31 | 19.0 | 0.0 |
| 1 | 20.7 | 0.0 |
| 2 | 20.2 | 0.0 |
| 3 | 21.2 | 0.0 |
| 4 | 21.5 | 0.0 |
| 5 | 22.8 | 0.0 |
| 6 | 24.8 | 5.3 |
| 7 | 23.1 | 0.0 |
| 8 | 19.0 | 5.0 |
| 9 | 19.6 | 0.0 |
| 10 | 18.5 | 0.0 |
| 11 | 18.8 | 0.9 |
| 12 | 20.0 | 0.0 |
| 13 | 21.0 | 0.0 |
| 14 | 22.4 | 0.0 |
| 15 | 22.0 | 0.0 |
| 16 | 21.2 | 4.0 |
| 17 | 22.8 | 11.0 |
| 18 | 19.7 | 0.0 |
| 19 | 19.3 | 0.0 |
| 20 | 19.2 | 0.0 |
| 21 | 20.0 | 25.6 |
| 22 | 15.3 | 0.6000000000000006 |
| 23 | 18.0 | 0.0 |
| 24 | 17.2 | 0.0 |
| 25 | 19.2 | 18.1 |
| 26 | 21.4 | 0.0 |
| 27 | 22.1 | 1.1 |
| 28 | 25.2 | 0.7000000000000006 |
| 29 | 20.1 | 1.7000000000000004 |
| 30 | 14.8 | 0.0 |
| 31 | 16.5 | 0.0 |
| 1 | 19.1 | 0.0 |
| 2 | 24.4 | 1.0 |
| 3 | 22.7 | 0.0 |
| 4 | 22.0 | 0.0 |
| 5 | 24.4 | 1.8 |
| 6 | 18.7 | 0.0 |
| 7 | 17.2 | 0.0 |
| 8 | 11.5 | 0.0 |
| 9 | 11.4 | 0.0 |
| 10 | 11.7 | 10.1 |
| 11 | 16.3 | 0.0 |
| 12 | 15.5 | 0.0 |
| 13 | 19.3 | 0.0 |
| 14 | 21.8 | 0.0 |
| 15 | 21.6 | 0.0 |
| 16 | 21.9 | 0.0 |
| 17 | 20.8 | 0.0 |
| 18 | 19.4 | 0.0 |
| 19 | 16.8 | 0.0 |
| 20 | 14.4 | 0.0 |
| 21 | 19.9 | 2.9 |
| 22 | 10.0 | 0.0 |
| 23 | 10.3 | 0.0 |
| 24 | 13.0 | 3.8 |
| 25 | 17.7 | 11.3 |
| 26 | 13.5 | 0.0 |
| 27 | 9.6 | 0.0 |
| 28 | 11.6 | 3.4 |
| 29 | 9.4 | 0.0 |
| 30 | 8.6 | 8.1 |
| 1 | 8.0 | 12.5 |
| 2 | 11.3 | 9.5 |
| 3 | 14.1 | 0.0 |
| 4 | 11.8 | 5.4 |
| 5 | 5.9 | 0.0 |
| 6 | 14.9 | 4.7 |
| 7 | 12.5 | 0.0 |
| 8 | 13.1 | 5.1 |
| 9 | 19.9 | 0.0 |
| 10 | 20.0 | 0.0 |
| 11 | 12.5 | 0.8 |
| 12 | 6.0 | 0.9 |
| 13 | 3.0 | 0.0 |
| 14 | 6.2 | 0.0 |
| 15 | 6.2 | 4.3 |
| 16 | 6.3 | 0.9 |
| 17 | 3.2 | 2.6 |
| 18 | 2.1 | 0.0 |
| 19 | 7.9 | 0.0 |
| 20 | 5.6 | 0.9 |
| 21 | 1.6 | 0.0 |
| 22 | 4.4 | 0.0 |
| 23 | 2.9 | 1.7000000000000004 |
| 24 | 0.9 | 0.0 |
| 25 | 3.7 | 0.0 |
| 26 | 3.1 | 0.0 |
| 27 | 1.9000000000000001 | 9.5 |
| 28 | 2.4 | 1.3 |
| 29 | 3.1 | 0.0 |
| 30 | 3.8 | 3.7 |
| 31 | 7.2 | 22.6 |C
----------------- 123 days --------------------
Harvest: 12.10.2018
Planting: 11.06.2018
Supplementary Figure S1
