# Supplementary material for: Effects of Nitrogen Application on Nitrogen Fixation in Common Bean Production
Source: Front Plant Sci. 2020 Aug 6;11:1172. doi: 10.3389/fpls.2020.01172 (PMC7424037; doi:10.3389/fpls.2020.01172)
Supplement: Supplementary file 2 [file Table_1.docx]

**Supplementary Table S1 |** Soil properties of trial sites used for nitrogen utilization experiments conducted at the Elora research station (ERS), ON in 2017 (ERS_2017) and 2018 (ERS_2018). N-poor soils were created by planting barley without nitrogen fertilizer in two consecutive years and harvesting the crop before maturity as a hay to ensure that all the top plant parts were removed from the fields. An Oakfield Classic Soil Probe was used to randomly collect soil samples at 0 to 22.9 cm depths across all areas of each N treatment within each replication to create bulk samples. After hand mixing, representative samples were sent for analysis to the SGS Agri-Food Laboratories Inc., Guelph, ON. Soil was not analyzed in 2016 pilot study.

| **Soil property** | | **ERS_2017** | | **ERS_2018** | |
| --- | --- | --- | --- | --- | --- |
|  |  | **N** | **NoN** | **N** | **NoN** |
| pH | | 7.4 | 7.6 | 7.4 | 7.5 |
| Organic matter (%) | | 2.7 | 2.5 | 3.5 | 3.5 |
| Nitrate (NO_3_^-^) N (ppm) | | 38.0 | 15.8 | 38.8 | 20.9 |
| Ammonium (NH_4_^+^) N (ppm) | | 1.0 | 0.9 | 7.1 | 2.7 |
| P (ppm) | | 22 | 18 | 30.9 | 30.1 |
| K (ppm) | | 82 | 79 | 104.3 | 99.3 |
| Mg (ppm) | | 338 | 312 | 389.6 | 394.9 |
| Ca (ppm) | | 2,675 | 2,655 | 2,697 | 2,636 |
| Zn (ppm) | | 1.0 | 1.0 | 1.9 | 1.8 |
| Zn Index | | 14.7 | 14.5 | 18.9 | 18.3 |
| Mn (ppm) | | 11.7 | 11.8 | 16.3 | 14.0 |
| Mn Index | | 15.0 | 16.5 | 16.3 | 15.9 |
| Cu (ppm) | | 1.2 | 1.2 | 1.8 | 1.8 |
| Fe (ppm) | | 18.1 | 16.8 | 30.3 | 30.7 |
| B (ppm) | | 0.47 | 0.44 | 0.6 | 0.6 |
| Texture^1^ | | M^1^ | M | M | M |
| CEC (meg 100g^-1^)^2^ | | 17.6 | 17.3 | 18.2 | 17.9 |
| Base saturation (%) | K | 1.2 | 1.2 | 1.5 | 1.4 |
|  | Mg | 16.0 | 15.0 | 17.8 | 18.4 |
|  | Ca | 76.0 | 76.8 | 74.1 | 73.5 |
|  | H | 6.8 | 6.9 | 6.6 | 6.8 |
| K/Mg ratio | | 0.2 | 0.3 | 0.3 | 0.3 |

^1^Medium or ‘Loamy’ texture; soils are classified as F (fine, for clay), C (coarse, for sand), M (for medium or ‘loamy’) and O (organic, for muck soils) texture; this is the technician’s interpretation by feel, not a measured value;

OMAFRA. (2017). Agronomy Guide for Field Crops Publication 811 (available at: http://www.omafra.gov.on.ca/english/crops/pub811/pub811.pdf; verified: 28 April 2020);

OMAFRA. (2018). Soil Fertility Handbook, Publication 611 (available at: http://www.omafra.gov.on.ca/english/crops/pub611/pub611.pdf; ISBN 978-1-4868-2402-1; verified 28 April 2020)

^2^Cation exchange capacity
