# Supplementary material for: Effects of Nitrogen Application on Nitrogen Fixation in Common Bean Production
Source: Front Plant Sci. 2020 Aug 6;11:1172. doi: 10.3389/fpls.2020.01172 (PMC7424037; doi:10.3389/fpls.2020.01172)
Supplement: Supplementary file 3 [file Table_2.docx]

**Supplementary Table S2 |** Random effects – 2016 experiment

**Percent nitrogen derived from atmosphere (%Ndfa, %) 2016**

**Covariance Parameter Estimates - %Ndfa 2016**

| **Cov Parm** | **Estimate** | **Standard Error** |
| --- | --- | --- |
| **Rep** | -28.1471 | 46.9181 |
| **Rep*Nitrogen** | 93.2844 | 85.5061 |
| **Rep*Nitrogen*Rhizobia** | 20.6030 | 12.9170 |
| **Residual** | 29.6169 | 2.9548 |

**Tests of Covariance Parameters Based on the Restricted Likelihood - %Ndfa 2016**

| **Label** | **DF** | **-2 Res Log Like** | **ChiSq** | **Pr > ChiSq** | **Note** |
| --- | --- | --- | --- | --- | --- |
| **Rep=0** | 1 | 1469.54 | 0.44 | 0.5075 | DF |
| **Rep*Nitrogen=0** | 1 | 1474.37 | 5.27 | 0.0217 | DF |
| **Rep*Rhizobia*Nitrogen=0** | 1 | 1515.82 | 46.72 | <.0001 | DF |

DF: P-value based on a chi-square with DF degrees of freedom.

Because estimation is unconstrained, likelihood-ratio tests are conducted as two-sided tests

**Maturity (DM, days) 2016**

**Covariance Parameter Estimates – DM 2016**

| **Cov Parm** | **Estimate** | **Standard Error** |
| --- | --- | --- |
| **Rep** | 0.1347 | 0.3024 |
| **Rep*Nitrogen** | -0.3423 | 0.5148 |
| **Rep*Nitrogen*Rhizobia** | 1.1471 | 0.8243 |
| **Residual** | 5.5118 | 0.5208 |

**Tests of Covariance Parameters Based on the Restricted Likelihood – DM 2016**

| **Label** | **DF** | **-2 Res Log Like** | **ChiSq** | **Pr > ChiSq** | **Note** |
| --- | --- | --- | --- | --- | --- |
| **Rep=0** | 1 | 1199.96 | 0.39 | 0.5312 | DF |
| **Rep*Nitrogen=0** | 1 | 1199.99 | 0.43 | 0.5115 | DF |
| **Rep*Rhizobia*Nitrogen=0** | 1 | 1215.03 | 15.47 | <.0001 | DF |

DF: P-value based on a chi-square with DF degrees of freedom.

Because estimation is unconstrained, likelihood-ratio tests are conducted as two-sided tests.

**Harvestability (HR, scale 1 to 5) 2016**

**Covariance Parameter Estimates – HR 2016**

| **Cov Parm** | **Estimate** | **Standard Error** |
| --- | --- | --- |
| **Rep** | 0.06110 | 0.05245 |
| **Rep*Nitrogen** | -0.01385 | 0.01257 |
| **Rep*Nitrogen*Rhizobia** | 0.02713 | 0.02316 |
| **Residual** | 0.2558 | 0.02412 |

| **Tests of Covariance Parameters Based on the Restricted Likelihood – HR 2016** | | | | | |
| --- | --- | --- | --- | --- | --- |
| **Label** | **DF** | **-2 Res Log Like** | **ChiSq** | **Pr > ChiSq** | **Note** |
| **Rep=0** | 1 | 480.35 | 5.25 | 0.0220 | DF |
| **Rep*Nitrogen=0** | 1 | 476.28 | 1.18 | 0.2768 | DF |
| **Rep*Rhizobia*Nitrogen=0** | 1 | 480.60 | 5.50 | 0.0190 | DF |

DF: P-value based on a chi-square with DF degrees of freedom.

Because estimation is unconstrained, likelihood-ratio tests are conducted as two-sided tests.

**Yield (YD, kg ha^-1^) 2016**

**Covariance Parameter Estimates – YD 2016**

| **Cov Parm** | **Estimate** | **Standard Error** |
| --- | --- | --- |
| **Rep** | -65.3711 | 6624.64 |
| **Rep*Nitrogen** | -3185.84 | 12691 |
| **Rep*Nitrogen*Rhizobia** | 24008 | 17011 |
| **Residual** | 108845 | 10194 |

**Tests of Covariance Parameters Based on the Restricted Likelihood – YD 2016**

| **Label** | **DF** | **-2 Res Log Like** | **ChiSq** | **Pr > ChiSq** | **Note** |
| --- | --- | --- | --- | --- | --- |
| **Rep=0** | 1 | 3594.30 | 0.09 | 0.7601 | DF |
| **Rep*Nitrogen=0** | 1 | 3594.30 | 0.09 | 0.7601 | DF |
| **Rep*Rhizobia*Nitrogen=0** | 1 | 3609.15 | 14.94 | 0.0001 | DF |

DF: P-value based on a chi-square with DF degrees of freedom.

Because estimation is unconstrained, likelihood-ratio tests are conducted as two-sided tests.

**Carbon isotope discrimination (δ^13^C , ‰) 2016**

**Covariance Parameter Estimates - δ^13^C**

| **Cov Parm** | **Estimate** | **Standard Error** |
| --- | --- | --- |
| **Rep** | -0.00023 | 0.005622 |
| **Rep*Nitrogen** | 0.002938 | 0.009069 |
| **Rep*Nitrogen*Rhizobia** | 0.008818 | 0.008106 |
| **Residual** | 0.1034 | 0.009727 |

**Tests of Covariance Parameters Based on the Restricted Likelihood - δ^13^C**

| **Label** | **DF** | **-2 Res Log Like** | **ChiSq** | **Pr > ChiSq** | **Note** |
| --- | --- | --- | --- | --- | --- |
| **Rep=0** | 1 | 259.54 | 0.00 | 0.9669 | DF |
| **Rep*Nitrogen=0** | 1 | 259.67 | 0.13 | 0.7216 | DF |
| **Rep*Rhizobia*Nitrogen=0** | 1 | 263.56 | 4.01 | 0.0451 | DF |

DF: P-value based on a chi-square with DF degrees of freedom.

Because estimation is unconstrained, likelihood-ratio tests are conducted as two-sided tests.
