# Supplementary material for: Effects of Nitrogen Application on Nitrogen Fixation in Common Bean Production
Source: Front Plant Sci. 2020 Aug 6;11:1172. doi: 10.3389/fpls.2020.01172 (PMC7424037; doi:10.3389/fpls.2020.01172)
Supplement: Supplementary file 6 [file Table_5.docx]

**Supplementary Table S5 |** Random effects – 2017_2018 combined experiment

**Percent nitrogen derived from atmosphere (%Ndfa, %) 2017_2018**

**Covariance Parameter Estimates - %Ndfa 2017_2018**

| **Cov Parm** | **Estimate** | **Standard Error** |
| --- | --- | --- |
| **Year** | 0.5162 | 4.0260 |
| **Rep(Year)** | 8.0345 | 5.2625 |
| **Rep*Rhizobia** | 0.4719 | 2.1106 |
| **Rep*Rhizobia*Nitrogen** | 2.5705 | 2.2260 |
| **Residual** | 52.4260 | 3.1731 |

**Tests of Covariance Parameters Based on the Restricted Likelihood - %Ndfa 2017_2018**

| **Label** | **DF** | **-2 Res Log Like** | **ChiSq** | **Pr > ChiSq** | **Note** |
| --- | --- | --- | --- | --- | --- |
| **Year=0** | 1 | 4020.40 | 0.02 | 0.4436 | MI |
| **Rep(Year)=0** | 1 | 4060.33 | 39.95 | <.0001 | MI |
| **Rep*Rhizobia=0** | 1 | 4020.43 | 0.05 | 0.4079 | MI |
| **Rep*Rhizobia*Nitrogen=0** | 1 | 4025.67 | 5.30 | 0.0107 | MI |

MI: P-value based on a mixture of chi-squares.

**Fowering (DF, days) 2017_2018**

**Covariance Parameter Estimates – DF 2017_2018**

| **Cov Parm** | **Estimate** | **Standard Error** |
| --- | --- | --- |
| **Year** | 48.1894 | 68.5380 |
| **Rep(Year)** | 0.04085 | 0.09563 |
| **Rep*Rhizobia** | 0.1509 | 0.1761 |
| **Rep*Rhizobia*Nitrogen** | 0.01257 | 0.04815 |
| **Residual** | 3.0754 | 0.1785 |

**Tests of Covariance Parameters Based on the Restricted Likelihood – DF 2017_2018**

| **Label** | **DF** | **-2 Res Log Like** | **ChiSq** | **Pr > ChiSq** | **Note** |
| --- | --- | --- | --- | --- | --- |
| **Year=0** | 1 | 2644.36 | 27.24 | <.0001 | MI |
| **Rep(Year)=0** | 1 | 2617.78 | 0.65 | 0.2096 | MI |
| **Rep*Rhizobia=0** | 1 | 2617.83 | 0.71 | 0.1997 | MI |
| **Rep*Rhizobia*Nitrogen=0** | 1 | 2617.21 | 0.08 | 0.3857 | MI |

MI: P-value based on a mixture of chi-squares.

**Maturity (DM, days) 2017_2018**

**Covariance Parameter Estimates – DM 2017_2018**

| **Cov Parm** | **Estimate** | **Standard Error** |
| --- | --- | --- |
| **Year** | 74.4725 | 108.87 |
| **Rep(Year)** | 2.0135 | 1.4385 |
| **Rep*Rhizobia** | 0.2021 | 0.4337 |
| **Rep*Rhizobia*Nitrogen** | 0.2861 | 0.2800 |
| **Residual** | 8.6540 | 0.5029 |

**Tests of Covariance Parameters Based on the Restricted Likelihood – DM 2017_2018**

| **Label** | **DF** | **-2 Res Log Like** | **ChiSq** | **Pr > ChiSq** | **Note** |
| --- | --- | --- | --- | --- | --- |
| **Year=0** | 1 | 3282.91 | 26.87 | <.0001 | DF |
| **Rep(Year)=0** | 1 | 3276.90 | 20.86 | <.0001 | DF |
| **Rep*Rhizobia=0** | 1 | 3256.39 | 0.35 | 0.5551 | DF |
| **Rep*Rhizobia*Nitrogen=0** | 1 | 3259.27 | 3.23 | 0.0722 | DF |

DF: P-value based on a chi-square with DF degrees of freedom.

Because estimation is unconstrained, likelihood-ratio tests are conducted as two-sided tests.

**Plant height (PH, cm) 2017_2018**

**Covariance Parameter Estimates – PH 2017_2018**

| **Cov Parm** | **Estimate** | **Standard Error** |
| --- | --- | --- |
| **Year** | 0.8588 | 2.6080 |
| **Rep(Year)** | 3.5242 | 2.2456 |
| **Rep*Rhizobia** | 0.7768 | 0.8850 |
| **Rep*Rhizobia*Nitrogen** | 0.3773 | 0.5005 |
| **Residual** | 21.2363 | 1.2315 |

**Tests of Covariance Parameters Based on the Restricted Likelihood – PH 2017_2018**

| **Label** | **DF** | **-2 Res Log Like** | **ChiSq** | **Pr > ChiSq** | **Note** |
| --- | --- | --- | --- | --- | --- |
| **Year=0** | 1 | 3812.27 | 0.21 | 0.3248 | MI |
| **Rep(Year)=0** | 1 | 3868.11 | 56.05 | <.0001 | MI |
| **Rep*Rhizobia=0** | 1 | 3813.36 | 1.29 | 0.1278 | MI |
| **Rep*Rhizobia*Nitrogen=0** | 1 | 3813.25 | 1.18 | 0.1383 | MI |

MI: P-value based on a mixture of chi-squares.

**Harvestability (HR, scale 1 to 5) 2017_2018**

**Covariance Parameter Estimates – HR 2017_2018**

| **Cov Parm** | **Estimate** | **Standard Error** |
| --- | --- | --- |
| **Year** | 0.000343 | 0.01653 |
| **Rep(Year)** | 0.03931 | 0.02387 |
| **Rep*Rhizobia** | -0.00330 | 0.002717 |
| **Rep*Rhizobia*Nitrogen** | 0.003065 | 0.005124 |
| **Residual** | 0.2535 | 0.01469 |

**Tests of Covariance Parameters Based on the Restricted Likelihood – HR 2017_2018**

| **Label** | **DF** | **-2 Res Log Like** | **ChiSq** | **Pr > ChiSq** | **Note** |
| --- | --- | --- | --- | --- | --- |
| **Year=0** | 1 | 1099.18 | 1.53 | 0.2167 | DF |
| **Rep(Year)=0** | 1 | 1136.28 | 38.63 | <.0001 | DF |
| **Rep*Rhizobia=0** | 1 | 1099.27 | 1.62 | 0.2033 | DF |
| **Rep*Rhizobia*Nitrogen=0** | 1 | 1099.27 | 1.62 | 0.2033 | DF |

DF: P-value based on a chi-square with DF degrees of freedom.

Because estimation is unconstrained, likelihood-ratio tests are conducted as two-sided tests.

**Yield (YD, kg ha^-1^) 2017_2018**

**Covariance Parameter Estimates – YD 2017_2018**

| **Cov Parm** | **Estimate** | **Standard Error** |
| --- | --- | --- |
| **Year** | 2554510 | 3852701 |
| **Rep(Year)** | 63381 | 47794 |
| **Rep*Rhizobia** | 15946 | 27827 |
| **Rep*Rhizobia*Nitrogen** | 26232 | 17181 |
| **Residual** | 153981 | 8972.56 |

**Tests of Covariance Parameters Based on the Restricted Likelihood – YD 2017_2018**

| **Label** | **DF** | **-2 Res Log Like** | **ChiSq** | **Pr > ChiSq** | **Note** |
| --- | --- | --- | --- | --- | --- |
| **Year=0** | 1 | 9193.00 | 15.46 | <.0001 | MI |
| **Rep(Year)=0** | 1 | 9249.39 | 71.84 | <.0001 | MI |
| **Rep*Rhizobia=0** | 1 | 9178.07 | 0.53 | 0.2336 | MI |
| **Rep*Rhizobia*Nitrogen=0** | 1 | 9207.51 | 29.97 | <.0001 | MI |

MI: P-value based on a mixture of chi-squares

**Seed weight (SW, g) 2017_2018**

**Covariance Parameter Estimates – SW 2017_2018**

| **Cov Parm** | **Estimate** | **Standard Error** |
| --- | --- | --- |
| **Year** | -0.00848 | 0.03004 |
| **Rep(Year)** | 0.08970 | 0.07775 |
| **Rep*Rhizobia** | 0.02545 | 0.08076 |
| **Rep*Rhizobia*Nitrogen** | 0.1092 | 0.07354 |
| **Residual** | 0.7755 | 0.04530 |

**Tests of Covariance Parameters Based on the Restricted Likelihood – SW 2017_2018**

| **Label** | **DF** | **-2 Res Log Like** | **ChiSq** | **Pr > ChiSq** | **Note** |
| --- | --- | --- | --- | --- | --- |
| **Year=0** | 1 | 1773.17 | 0.06 | 0.8029 | DF |
| **Rep(Year)=0** | 1 | 1792.38 | 19.27 | <.0001 | DF |
| **Rep*Rhizobia=0** | 1 | 1773.31 | 0.20 | 0.6516 | DF |
| **Rep*Rhizobia*Nitrogen=0** | 1 | 1796.75 | 23.64 | <.0001 | DF |

DF: P-value based on a chi-square with DF degrees of freedom.

Because estimation is unconstrained, likelihood-ratio tests are conducted as two-sided tests.

**Leaf chlorophyll content (SPAD, SPAD values) 2017_2018**

**Covariance Parameter Estimates – SPAD 2017_2018**

| **Cov Parm** | **Estimate** | **Standard Error** |
| --- | --- | --- |
| **Year** | 0.5368 | 0.7853 |
| **Rep(Year)** | 0.01752 | 0.06863 |
| **Rep*Rhizobia** | 0.2714 | 0.3170 |
| **Rep*Rhizobia*Nitrogen** | 0.2924 | 0.2322 |
| **Residual** | 4.8126 | 0.2794 |

**Tests of Covariance Parameters Based on the Restricted Likelihood – SPAD 2017_2018**

| **Label** | **DF** | **-2 Res Log Like** | **ChiSq** | **Pr > ChiSq** | **Note** |
| --- | --- | --- | --- | --- | --- |
| **Year=0** | 1 | 2896.38 | 3.92 | 0.0478 | DF |
| **Rep(Year)=0** | 1 | 2892.55 | 0.10 | 0.7561 | DF |
| **Rep*Rhizobia=0** | 1 | 2893.32 | 0.86 | 0.3533 | DF |
| **Rep*Rhizobia*Nitrogen=0** | 1 | 2900.44 | 7.98 | 0.0047 | DF |

DF: P-value based on a chi-square with DF degrees of freedom.

Because estimation is unconstrained, likelihood-ratio tests are conducted as two-sided tests.

**Carbon isotope discrimination (δ^13^C) 2017_2018**

**Covariance Parameter Estimates - δ^13^C 2017_2018**

| **Cov Parm** | **Estimate** | **Standard Error** |
| --- | --- | --- |
| **Year** | -0.00483 | 0.009174 |
| **Rep(Year)** | 0.03680 | 0.02397 |
| **Rep*Rhizobia** | -0.00153 | 0.006269 |
| **Rep*Rhizobia*Nitrogen** | 0.009943 | 0.008250 |
| **Residual** | 0.1864 | 0.01088 |

**Tests of Covariance Parameters Based on the Restricted Likelihood - δ^13^C 2017_2018**

| **Label** | **DF** | **-2 Res Log Like** | **ChiSq** | **Pr > ChiSq** | **Note** |
| --- | --- | --- | --- | --- | --- |
| **Year=0** | 1 | 909.58 | 0.24 | 0.6255 | DF |
| **Rep(Year)=0** | 1 | 954.66 | 45.32 | <.0001 | DF |
| **Rep*Rhizobia=0** | 1 | 909.59 | 0.25 | 0.6196 | DF |
| **Rep*Rhizobia*Nitrogen=0** | 1 | 915.99 | 6.64 | 0.0099 | DF |

DF: P-value based on a chi-square with DF degrees of freedom.

Because estimation is unconstrained, likelihood-ratio tests are conducted as two-sided tests

**Supplementary Table S5-1 |** Type III tests of fixed effects from restricted likelihood analysis of split-split plot experiments with 22 common bean genotypes conducted at the Elora research station (ON, Canada) on created K-P land in 2017.

| **Traits^1^** | **Effect** | **Numerator DF** | **Denominator DF** | **F Value** | **Pr > F** |
| --- | --- | --- | --- | --- | --- |
| %Ndfa^NB2^ | Rhizobia (R) | 1 | 3 | 3.13 | 0.1751 |
|  | Nitrogen (N) | 1 | 6.062 | 197.74 | <.0001 |
|  | R*N | 1 | 6.062 | 14.33 | 0.0089 |
|  | Genotype (G) | 20 | 220.3 | 3.43 | <.0001 |
|  | R*G | 20 | 220.3 | 1.37 | 0.1397 |
|  | N*G | 20 | 220.3 | 0.69 | 0.8308 |
|  | R*N*G | 20 | 220.3 | 0.83 | 0.6762 |
| YD^NB^ | Rhizobia (R) | 1 | 2.999 | 0.06 | 0.8165 |
|  | Nitrogen (N) | 1 | 5.982 | 14.65 | 0.0087 |
|  | R*N | 1 | 5.982 | 0.56 | 0.4825 |
|  | Genotype (G) | 21 | 248 | 11.46 | <.0001 |
|  | R*G | 21 | 248 | 0.84 | 0.6730 |
|  | N*G | 21 | 248 | 1.24 | 0.2197 |
|  | R*N*G | 21 | 248 | 0.83 | 0.6820 |
| δ^13^C | Rhizobia (R) | 1 | 2.993 | 0.02 | 0.9050 |
|  | Nitrogen (N) | 1 | 6.119 | 19.32 | 0.0044 |
|  | R*N | 1 | 6.119 | 0.07 | 0.7971 |
|  | Genotype (G) | 21 | 245.4 | 14.30 | <.0001 |
|  | R*G | 21 | 245.4 | 2.36 | 0.0010 |
|  | N*G | 21 | 245.4 | 1.37 | 0.1357 |
|  | R*N*G | 21 | 245.4 | 1.13 | 0.3185 |
| DF^NB^ | Rhizobia (R) | 1 | 2.992 | 0.32 | 0.6118 |
|  | Nitrogen (N) | 1 | 6.056 | 12.12 | 0.0129 |
|  | R*N | 1 | 6.057 | 0.64 | 0.4529 |
|  | Genotype (G) | 21 | 248.1 | 33.56 | <.0001 |
|  | R*G | 21 | 248.1 | 1.30 | 0.1742 |
|  | N*G | 21 | 248.1 | 1.13 | 0.3138 |
|  | R*N*G | 21 | 248.1 | 0.44 | 0.9861 |
| DM^NB^ | Rhizobia (R) | 1 | 2.998 | 1.75 | 0.2778 |
|  | Nitrogen (N) | 1 | 5.787 | 218.34 | <.0001 |
|  | R*N | 1 | 5.788 | 4.21 | 0.0879 |
|  | Genotype (G) | 21 | 246.1 | 27.87 | <.0001 |
|  | R*G | 21 | 246.1 | 0.96 | 0.5098 |
|  | N*G | 21 | 246.1 | 1.10 | 0.3479 |
|  | R*N*G | 21 | 246.1 | 1.00 | 0.4613 |
| PH | Rhizobia (R) | 1 | 2.999 | 0.06 | 0.8168 |
|  | Nitrogen (N) | 1 | 6.004 | 29.45 | 0.0016 |
|  | R*N | 1 | 6.004 | 0.03 | 0.8642 |
|  | Genotype (G) | 21 | 249 | 7.75 | <.0001 |
|  | R*G | 21 | 249 | 1.05 | 0.4017 |
|  | N*G | 21 | 249 | 0.77 | 0.7585 |
|  | R*N*G | 21 | 249 | 0.86 | 0.6458 |
| HR^NB^ | Rhizobia (R) | 1 | 2.749 | 2.78 | 0.2026 |
|  | Nitrogen (N) | 1 | 6.015 | 0.00 | 0.9983 |
|  | R*N | 1 | 6.015 | 1.34 | 0.2902 |
|  | Genotype (G) | 21 | 244.4 | 12.82 | <.0001 |
|  | R*G | 21 | 244.4 | 1.00 | 0.4617 |
|  | N*G | 21 | 244.5 | 1.92 | 0.0103 |
|  | R*N*G | 21 | 244.5 | 0.58 | 0.9314 |
| SW^NB^ | Rhizobia (R) | 1 | 2.999 | 0.16 | 0.7154 |
|  | Nitrogen (N) | 1 | 5.996 | 3.16 | 0.1261 |
|  | R*N | 1 | 5.996 | 0.00 | 0.9815 |
|  | Genotype (G) | 21 | 244.1 | 50.82 | <.0001 |
|  | R*G | 21 | 244.1 | 0.69 | 0.8420 |
|  | N*G | 21 | 244.1 | 2.88 | <.0001 |
|  | R*N*G | 21 | 244.1 | 0.60 | 0.9189 |
| SPAD^NB^ | Rhizobia (R) | 1 | 2.447 | 84.85 | 0.0059 |
|  | Nitrogen (N) | 1 | 6.032 | 2.05 | 0.2017 |
|  | R*N | 1 | 6.021 | 0.13 | 0.7352 |
|  | Genotype (G) | 21 | 247.5 | 22.31 | <.0001 |
|  | R*G | 21 | 247.5 | 1.16 | 0.2850 |
|  | N*G | 21 | 247.5 | 3.89 | <.0001 |
|  | R*N*G | 21 | 247.5 | 1.68 | 0.03 |

^1^%Ndfa, percent nitrogen derived from atmosphere (%); YD, yield (kg ha^-1^); δ^13^C, carbon isotope discrimination (‰); DF, flowering (days); DM, maturity (days); PH, plant height (cm); HR, harvestability (scale 1 to 5; SW, seed weight (g); SPAD, leaf chlorophyll content (SPAD units). Non nodulating genotype R99 was removed from the %Ndfa analysis.

^2^Glimmix procedure, 2017; Normal distribution, ddfm=kr (NB, nobound option)

**Supplementary Table S5-2 |** Type III tests of fixed effects from restricted likelihood analysis of split-split plot experiments with 22 common bean genotypes conducted at the Elora research station (ON, Canada) on created K-P land in 2018.

| **Trait^1^** | **Effect** | **Numerator DF** | **Denominator DF** | **F Value** | **Pr > F** |
| --- | --- | --- | --- | --- | --- |
| %Ndfa^NB2^ | Rhizobia (R) | 1 | 2.92 | 10.12 | 0.0519 |
|  | Nitrogen (N) | 1 | 5.842 | 292.14 | <.0001 |
|  | R*N | 1 | 5.844 | 1.30 | 0.2990 |
|  | Genotype (G) | 20 | 233.7 | 11.35 | <.0001 |
|  | R*G | 20 | 233.7 | 1.08 | 0.3702 |
|  | N*G | 20 | 233.7 | 1.22 | 0.2402 |
|  | R*N*G | 20 | 233.7 | 1.06 | 0.3970 |
| YD | Rhizobia (R) | 1 | 2.993 | 2.00 | 0.2520 |
|  | Nitrogen (N) | 1 | 6.025 | 17.04 | 0.0061 |
|  | R*N | 1 | 6.025 | 1.23 | 0.3092 |
|  | Genotype (G) | 21 | 245.1 | 16.90 | <.0001 |
|  | R*G | 21 | 245.1 | 2.10 | 0.0041 |
|  | N*G | 21 | 245.1 | 1.62 | 0.0451 |
|  | R*N*G | 21 | 245.1 | 1.45 | 0.0963 |
| δ^13^C^NB^ | Rhizobia (R) | 1 | 2.999 | 7.27 | 0.0740 |
|  | Nitrogen (N) | 1 | 6.006 | 0.02 | 0.8959 |
|  | R*N | 1 | 6.006 | 1.10 | 0.3341 |
|  | Genotype (G) | 21 | 248.1 | 10.95 | <.0001 |
|  | R*G | 21 | 248.1 | 1.36 | 0.1398 |
|  | N*G | 21 | 248.1 | 1.13 | 0.3197 |
|  | R*N*G | 21 | 248.1 | 1.27 | 0.1984 |
| DF^NB^ | Rhizobia (R) | 1 | 3.038 | 18.50 | 0.0225 |
|  | Nitrogen (N) | 1 | 5.966 | 12.43 | 0.0126 |
|  | R*N | 1 | 5.964 | 1.69 | 0.2413 |
|  | Genotype (G) | 21 | 250.2 | 17.93 | <.0001 |
|  | R*G | 21 | 250.2 | 1.18 | 0.2679 |
|  | N*G | 21 | 250.2 | 1.44 | 0.1009 |
|  | R*N*G | 21 | 250.2 | 1.16 | 0.2893 |
| DM^NB^ | Rhizobia (R) | 1 | 3.01 | 3.59 | 0.1542 |
|  | Nitrogen (N) | 1 | 6.016 | 10.56 | 0.0174 |
|  | R*N | 1 | 6.016 | 0.01 | 0.9264 |
|  | Genotype (G) | 21 | 251.1 | 23.36 | <.0001 |
|  | R*G | 21 | 251.1 | 1.21 | 0.2393 |
|  | N*G | 21 | 251.1 | 0.76 | 0.7636 |
|  | R*N*G | 21 | 251.1 | 0.90 | 0.5871 |
| PH | Rhizobia (R) | 1 | 2.997 | 0.27 | 0.6405 |
|  | Nitrogen (N) | 1 | 6.072 | 16.93 | 0.0061 |
|  | R*N | 1 | 6.072 | 2.91 | 0.1381 |
|  | Genotype (G) | 21 | 250.2 | 15.50 | <.0001 |
|  | R*G | 21 | 250.2 | 1.17 | 0.2761 |
|  | N*G | 21 | 250.2 | 1.16 | 0.2861 |
|  | R*N*G | 21 | 250.2 | 2.05 | 0.0052 |
| HR | Rhizobia (R) | 1 | 2.992 | 11.09 | 0.0449 |
|  | Nitrogen (N) | 1 | 6.027 | 32.61 | 0.0012 |
|  | R*N | 1 | 6.027 | 1.55 | 0.2591 |
|  | Genotype (G) | 21 | 251.2 | 16.07 | <.0001 |
|  | R*G | 21 | 251.2 | 0.37 | 0.9950 |
|  | N*G | 21 | 251.2 | 1.74 | 0.0258 |
|  | R*N*G | 21 | 251.2 | 1.45 | 0.0978 |
| SW | Rhizobia (R) | 1 | 2.983 | 1.05 | 0.3807 |
|  | Nitrogen (N) | 1 | 5.99 | 7.80 | 0.0315 |
|  | R*N | 1 | 5.99 | 0.37 | 0.5669 |
|  | Genotype (G) | 21 | 248 | 50.31 | <.0001 |
|  | R*G | 21 | 248 | 1.50 | 0.0776 |
|  | N*G | 21 | 248 | 1.33 | 0.1566 |
|  | R*N*G | 21 | 248 | 0.69 | 0.8383 |
| SPAD | Rhizobia (R) | 1 | 3.003 | 11.07 | 0.0448 |
|  | Nitrogen (N) | 1 | 6.022 | 0.04 | 0.8530 |
|  | R*N | 1 | 6.022 | 0.09 | 0.7714 |
|  | Genotype (G) | 21 | 251.1 | 9.56 | <.0001 |
|  | R*G | 21 | 251.1 | 0.94 | 0.5453 |
|  | N*G | 21 | 251.1 | 0.93 | 0.5551 |
|  | R*N*G | 21 | 251.1 | 0.60 | 0.9200 |

^1^%Ndfa, percent nitrogen derived from atmosphere (%); YD, yield (kg ha^-1^); δ^13^C, carbon isotope discrimination (‰); DF, flowering (days); DM, maturity (days); PH, plant height (cm); HR, harvestability (scale 1 to 5; SW, seed weight (g); SPAD, leaf chlorophyll content (SPAD units). Non nodulating genotype R99 was removed from the %Ndfa analysis.

^2^Glimmix procedure, 2018; Normal distribution, ddfm=kr (NB, nobound option)

**Supplementary Table S5-3 |** Random effects – 2017

**Percent nitrogen derived from atmosphere (%Ndfa, %) 20178**

**Covariance Parameter Estimates - %Ndfa 2017**

| **Cov Parm** | **Estimate** | **Standard Error** |
| --- | --- | --- |
| **Rep** | -2.3127 | 6.5310 |
| **Rep*Rhizobia** | 6.3472 | 11.6792 |
| **Rep*Rhizobia*Nitrogen** | 11.8852 | 8.1169 |
| **Residual** | 42.4140 | 4.0437 |

**Tests of Covariance Parameters Based on the Restricted Likelihood - %Ndfa 2017**

| **Label** | **DF** | **-2 Res Log Like** | **ChiSq** | **Pr > ChiSq** | **Note** |
| --- | --- | --- | --- | --- | --- |
| **Rep=0** | 1 | 1662.97 | 0.13 | 0.7143 | DF |
| **Rep*Rhizobia=0** | 1 | 1663.27 | 0.44 | 0.5070 | DF |
| **Rep*Rhizobia*Nitrogen=0** | 1 | 1682.54 | 19.70 | <.0001 | DF |

DF: P-value based on a chi-square with DF degrees of freedom.

Because estimation is unconstrained, likelihood-ratio tests are conducted as two-sided tests.

**Flowering (DF, days) 2017**

**Covariance Parameter Estimates – DF 2017**

| **Cov Parm** | **Estimate** | **Standard Error** |
| --- | --- | --- |
| **Rep** | 0.2348 | 0.2397 |
| **Rep*Rhizobia** | -0.00743 | 0.1107 |
| **Rep*Rhizobia*Nitrogen** | 0.07696 | 0.1339 |
| **Residual** | 3.3549 | 0.3013 |

**Tests of Covariance Parameters Based on the Restricted Likelihood – DF 2017**

| **Label** | **DF** | **-2 Res Log Like** | **ChiSq** | **Pr > ChiSq** | **Note** |
| --- | --- | --- | --- | --- | --- |
| **Rep=0** | 1 | 1184.76 | 1.90 | 0.1677 | DF |
| **Rep*Rhizobia=0** | 1 | 1182.87 | 0.00 | 0.9472 | DF |
| **Rep*Rhizobia*Nitrogen=0** | 1 | 1183.41 | 0.55 | 0.4579 | DF |

DF: P-value based on a chi-square with DF degrees of freedom.

Because estimation is unconstrained, likelihood-ratio tests are conducted as two-sided tests.

**Maturity (DM, days) 2017**

**Covariance Parameter Estimates – DM 2017**

| **Cov Parm** | **Estimate** | **Standard Error** |
| --- | --- | --- |
| **Rep** | 0.3904 | 0.4456 |
| **Rep*Rhizobia** | 0.2199 | 0.2274 |
| **Rep*Rhizobia*Nitrogen** | -0.02902 | 0.06704 |
| **Residual** | 3.0326 | 0.2736 |

**Tests of Covariance Parameters Based on the Restricted Likelihood – DM 2017**

| **Label** | **DF** | **-2 Res Log Like** | **ChiSq** | **Pr > ChiSq** | **Note** |
| --- | --- | --- | --- | --- | --- |
| **Rep=0** | 1 | 1150.95 | 1.45 | 0.2290 | DF |
| **Rep*Rhizobia=0** | 1 | 1152.24 | 2.73 | 0.0983 | DF |
| **Rep*Rhizobia*Nitrogen=0** | 1 | 1149.64 | 0.14 | 0.7079 | DF |

DF: P-value based on a chi-square with DF degrees of freedom.

Because estimation is unconstrained, likelihood-ratio tests are conducted as two-sided tests.

**Plant height (PH, cm) 2017**

**Covariance Parameter Estimates – PH 2017**

| **Cov Parm** | **Estimate** | **Standard Error** |
| --- | --- | --- |
| **Rep** | 2.5595 | 4.3572 |
| **Rep*Rhizobia** | 2.7410 | 3.8446 |
| **Rep*Rhizobia*Nitrogen** | 2.9144 | 2.0729 |
| **Residual** | 14.6940 | 1.3169 |

**Tests of Covariance Parameters Based on the Restricted Likelihood – PH 2017**

| **Label** | **DF** | **-2 Res Log Like** | **ChiSq** | **Pr > ChiSq** | **Note** |
| --- | --- | --- | --- | --- | --- |
| **Rep=0** | 1 | 1591.53 | 0.42 | 0.2591 | MI |
| **Rep*Rhizobia=0** | 1 | 1592.03 | 0.92 | 0.1682 | MI |
| **Rep*Rhizobia*Nitrogen=0** | 1 | 1605.77 | 14.66 | <.0001 | MI |

MI: P-value based on a mixture of chi-squares.

**Harvestability (HR, scale 1 to 5) 2017**

**Covariance Parameter Estimates – HR 2017**

| **Cov Parm** | **Estimate** | **Standard Error** |
| --- | --- | --- |
| **Rep** | -0.00085 | 0.000795 |
| **Rep*Rhizobia** | -0.01367 | 0.009155 |
| **Rep*Rhizobia*Nitrogen** | 0.02037 | 0.01804 |
| **Residual** | 0.2372 | 0.02150 |

**Tests of Covariance Parameters Based on the Restricted Likelihood – HR 2017**

| **Label** | **DF** | **-2 Res Log Like** | **ChiSq** | **Pr > ChiSq** | **Note** |
| --- | --- | --- | --- | --- | --- |
| **Rep=0** | 1 | 491.49 | 9.61 | 0.0019 | DF |
| **Rep*Rhizobia=0** | 1 | 491.48 | 9.60 | 0.0019 | DF |
| **Rep*Rhizobia*Nitrogen=0** | 1 | 492.92 | 11.04 | 0.0009 | DF |

DF: P-value based on a chi-square with DF degrees of freedom.

Because estimation is unconstrained, likelihood-ratio tests are conducted as two-sided tests.

**Yield (YD, kg ha^-1^) 2017**

**Covariance Parameter Estimates – YD 2017**

| **Cov Parm** | **Estimate** | **Standard Error** |
| --- | --- | --- |
| **Rep** | -5051.47 | 36054 |
| **Rep*Rhizobia** | 29553 | 59049 |
| **Rep*Rhizobia*Nitrogen** | 70531 | 43587 |
| **Residual** | 104885 | 9419.29 |

**Tests of Covariance Parameters Based on the Restricted Likelihood – YD 2017**

| **Label** | **DF** | **-2 Res Log Like** | **ChiSq** | **Pr > ChiSq** | **Note** |
| --- | --- | --- | --- | --- | --- |
| **Rep=0** | 1 | 3900.40 | 0.02 | 0.8880 | DF |
| **Rep*Rhizobia=0** | 1 | 3900.73 | 0.35 | 0.5524 | DF |
| **Rep*Rhizobia*Nitrogen=0** | 1 | 3957.62 | 57.24 | <.0001 | DF |

DF: P-value based on a chi-square with DF degrees of freedom.

Because estimation is unconstrained, likelihood-ratio tests are conducted as two-sided tests.

**Seed weight (SW, g) 2017**

**Covariance Parameter Estimates – SW 2017**

| **Cov Parm** | **Estimate** | **Standard Error** |
| --- | --- | --- |
| **Rep** | -0.08484 | 0.09595 |
| **Rep*Rhizobia** | 0.07905 | 0.2047 |
| **Rep*Rhizobia*Nitrogen** | 0.2751 | 0.1715 |
| **Residual** | 0.4675 | 0.04232 |

**Tests of Covariance Parameters Based on the Restricted Likelihood – SW 2017**

| **Label** | **DF** | **-2 Res Log Like** | **ChiSq** | **Pr > ChiSq** | **Note** |
| --- | --- | --- | --- | --- | --- |
| **Rep=0** | 1 | 682.59 | 1.33 | 0.2492 | DF |
| **Rep*Rhizobia=0** | 1 | 682.57 | 1.31 | 0.2525 | DF |
| **Rep*Rhizobia*Nitrogen=0** | 1 | 732.51 | 51.25 | <.0001 | DF |

DF: P-value based on a chi-square with DF degrees of freedom.

Because estimation is unconstrained, likelihood-ratio tests are conducted as two-sided tests.

**Leaf chlorophyll content (SPAD, SPAD values) 2017**

**Covariance Parameter Estimates – SPAD 2017**

| **Cov Parm** | **Estimate** | **Standard Error** |
| --- | --- | --- |
| **Rep** | 0.4025 | 0.3312 |
| **Rep*Rhizobia** | -0.1755 | 0.1044 |
| **Rep*Rhizobia*Nitrogen** | 0.2252 | 0.2091 |
| **Residual** | 2.9646 | 0.2671 |

**Tests of Covariance Parameters Based on the Restricted Likelihood – SPAD 2017**

| **Label** | **DF** | **-2 Res Log Like** | **ChiSq** | **Pr > ChiSq** | **Note** |
| --- | --- | --- | --- | --- | --- |
| **Rep=0** | 1 | 1153.39 | 10.55 | 0.0012 | DF |
| **Rep*Rhizobia=0** | 1 | 1149.52 | 6.68 | 0.0097 | DF |
| **Rep*Rhizobia*Nitrogen=0** | 1 | 1151.41 | 8.56 | 0.0034 | DF |

DF: P-value based on a chi-square with DF degrees of freedom.

Because estimation is unconstrained, likelihood-ratio tests are conducted as two-sided tests.

**Carbon isotope discrimination (δ^13^C, ‰) 2017**

**Covariance Parameter Estimates - δ^13^C 2017**

| **Cov Parm** | **Estimate** | **Standard Error** |
| --- | --- | --- |
| **Rep** | 0.08414 | 0.07226 |
| **Rep*Rhizobia** | 0.005037 | 0.007249 |
| **Rep*Rhizobia*Nitrogen** | 0.000288 | 0.004032 |
| **Residual** | 0.1434 | 0.01296 |

**Tests of Covariance Parameters Based on the Restricted Likelihood - δ^13^C 2017**

| **Label** | **DF** | **-2 Res Log Like** | **ChiSq** | **Pr > ChiSq** | **Note** |
| --- | --- | --- | --- | --- | --- |
| **Rep=0** | 1 | 370.44 | 5.22 | 0.0112 | MI |
| **Rep*Rhizobia=0** | 1 | 366.08 | 0.86 | 0.1767 | MI |
| **Rep*Rhizobia*Nitrogen=0** | 1 | 365.23 | 0.01 | 0.4708 | MI |

MI: P-value based on a mixture of chi-squares.

**Supplementary Table S5-4 |** Random effects – 2018 combined experiment

**Percent nitrogen derived from atmosphere (%Ndfa, %) 2018 (NB)**

**Covariance Parameter Estimates - %Ndfa 2018**

| **Cov Parm** | **Estimate** | **Standard Error** |
| --- | --- | --- |
| **Rep** | 13.6947 | 11.5322 |
| **Rep*Rhizobia** | -0.9811 | 1.2477 |
| **Rep*Rhizobia*Nitrogen** | 2.3756 | 2.1243 |
| **Residual** | 25.5784 | 2.3676 |

**Tests of Covariance Parameters Based on the Restricted Likelihood - %Ndfa 2018**

| **Label** | **DF** | **-2 Res Log Like** | **ChiSq** | **Pr > ChiSq** | **Note** |
| --- | --- | --- | --- | --- | --- |
| **Rep=0** | 1 | 1635.55 | 6.57 | 0.0104 | DF |
| **Rep*Rhizobia=0** | 1 | 1629.53 | 0.55 | 0.4593 | DF |
| **Rep*Rhizobia*Nitrogen=0** | 1 | 1633.52 | 4.54 | 0.0331 | DF |

DF: P-value based on a chi-square with DF degrees of freedom.

Because estimation is unconstrained, likelihood-ratio tests are conducted as two-sided tests.

**Flowering (DF 2018**

**Covariance Parameter Estimates – DF 2018**

| **Cov Parm** | **Estimate** | **Standard Error** |
| --- | --- | --- |
| **Rep** | 0.1768 | 0.1503 |
| **Rep*Rhizobia** | -0.00149 | 0.01471 |
| **Rep*Rhizobia*Nitrogen** | 0.005136 | 0.01836 |
| **Residual** | 0.5744 | 0.05137 |

**Tests of Covariance Parameters Based on the Restricted Likelihood – DF 2018**

| **Label** | **DF** | **-2 Res Log Like** | **ChiSq** | **Pr > ChiSq** | **Note** |
| --- | --- | --- | --- | --- | --- |
| **Rep=0** | 1 | 737.07 | 6.09 | 0.0136 | DF |
| **Rep*Rhizobia=0** | 1 | 730.99 | 0.01 | 0.9211 | DF |
| **Rep*Rhizobia*Nitrogen=0** | 1 | 731.08 | 0.10 | 0.7542 | DF |

DF: P-value based on a chi-square with DF degrees of freedom.

Because estimation is unconstrained, likelihood-ratio tests are conducted as two-sided tests.

**Maturity (DM, days) 2018**

**Covariance Parameter Estimates – DM 2018**

| **Cov Parm** | **Estimate** | **Standard Error** |
| --- | --- | --- |
| **Rep** | 4.2187 | 3.6972 |
| **Rep*Rhizobia** | -0.3073 | 0.7113 |
| **Rep*Rhizobi*Nitrogen** | 1.3021 | 1.0435 |
| **Residual** | 11.0868 | 0.9896 |

**Tests of Covariance Parameters Based on the Restricted Likelihood – DM 2018**

| **Label** | **DF** | **-2 Res Log Like** | **ChiSq** | **Pr > ChiSq** | **Note** |
| --- | --- | --- | --- | --- | --- |
| **Rep=0** | 1 | 1526.08 | 4.38 | 0.0364 | DF |
| **Rep*Rhizobia=0** | 1 | 1521.87 | 0.16 | 0.6846 | DF |
| **Rep*Rhizobia*Nitrogen=0** | 1 | 1529.11 | 7.40 | 0.0065 | DF |

DF: P-value based on a chi-square with DF degrees of freedom.

Because estimation is unconstrained, likelihood-ratio tests are conducted as two-sided test

**Plant height (PH, cm) 2018**

**Covariance Parameter Estimates – PH 2018**

| **Cov Parm** | **Estimate** | **Standard Error** |
| --- | --- | --- |
| **Rep** | 1.5825 | 1.9773 |
| **Rep*Rhizobia** | 0.9257 | 1.2285 |
| **Rep*Rhizobia*Nitrogen** | 0.3661 | 0.6118 |
| **Residual** | 15.1651 | 1.3561 |

**Tests of Covariance Parameters Based on the Restricted Likelihood – PH 2018**

| **Label** | **DF** | **-2 Res Log Like** | **ChiSq** | **Pr > ChiSq** | **Note** |
| --- | --- | --- | --- | --- | --- |
| **Rep=0** | 1 | 1592.86 | 0.95 | 0.1650 | MI |
| **Rep*Rhizobia=0** | 1 | 1593.01 | 1.10 | 0.1473 | MI |
| **Rep*Rhizobia*Nitrogen=0** | 1 | 1592.52 | 0.61 | 0.2168 | MI |

MI: P-value based on a mixture of chi-squares.

**Harvestability (HR, scale 1 to 5) 2018**

**Covariance Parameter Estimates – HR 2018**

| **Cov Parm** | **Estimate** | **Standard Error** |
| --- | --- | --- |
| **Rep** | 0.07829 | 0.06672 |
| **Rep*Rhizobia** | 0.001486 | 0.006212 |
| **Rep*Rhizobia*Nitrogen** | 0.000598 | 0.006006 |
| **Residual** | 0.2131 | 0.01902 |

**Tests of Covariance Parameters Based on the Restricted Likelihood – HR 2018**

| **Label** | **DF** | **-2 Res Log Like** | **ChiSq** | **Pr > ChiSq** | **Note** |
| --- | --- | --- | --- | --- | --- |
| **Rep=0** | 1 | 478.94 | 5.67 | 0.0086 | MI |
| **Rep*Rhizobia=0** | 1 | 473.33 | 0.07 | 0.3992 | MI |
| **Rep*Rhizobia*Nitrogen=0** | 1 | 473.27 | 0.01 | 0.4588 | MI |

MI: P-value based on a mixture of chi-squares.

**Yield (YD, kg ha^-1^) 2018**

**Covariance Parameter Estimates – YD 2018**

| **Cov Parm** | **Estimate** | **Standard Error** |
| --- | --- | --- |
| **Rep** | 129595 | 118364 |
| **Rep*Rhizobia** | 16843 | 24913 |
| **Rep*Rhizobia*Nitrogen** | 18325 | 14291 |
| **Residual** | 138334 | 12499 |

**Tests of Covariance Parameters Based on the Restricted Likelihood – YD 2018**

| **Label** | **DF** | **-2 Res Log Like** | **ChiSq** | **Pr > ChiSq** | **Note** |
| --- | --- | --- | --- | --- | --- |
| **Rep=0** | 1 | 3922.95 | 3.29 | 0.0349 | MI |
| **Rep*Rhizobia=0** | 1 | 3920.46 | 0.79 | 0.1868 | MI |
| **Rep*Rhizobia*Nitrogen=0** | 1 | 3928.16 | 8.50 | 0.0018 | MI |

MI: P-value based on a mixture of chi-squares.

**Seed weight (SW, g) 2018**

**Covariance Parameter Estimates – SW 2018**

| **Cov Parm** | **Estimate** | **Standard Error** |
| --- | --- | --- |
| **Rep** | 0.2246 | 0.2152 |
| **Rep*Rhizobia** | 0.02786 | 0.06473 |
| **Rep*Rhizobia*Nitrogen** | 0.05291 | 0.05197 |
| **Residual** | 0.7981 | 0.07169 |

**Tests of Covariance Parameters Based on the Restricted Likelihood – SW 2018**

| **Label** | **DF** | **-2 Res Log Like** | **ChiSq** | **Pr > ChiSq** | **Note** |
| --- | --- | --- | --- | --- | --- |
| **Rep=0** | 1 | 822.08 | 2.53 | 0.0558 | MI |
| **Rep*Rhizobia=0** | 1 | 819.80 | 0.25 | 0.3102 | MI |
| **Rep*Rhizobia*Nitrogen=0** | 1 | 822.67 | 3.12 | 0.0387 | MI |

MI: P-value based on a mixture of chi-squares.

**Leaf chlorophyll content (SPAD, SPAD values) 2018**

**Covariance Parameter Estimates – SPAD 2018**

| **Cov Parm** | **Estimate** | **Standard Error** |
| --- | --- | --- |
| **Rep** | 0.2465 | 0.5303 |
| **Rep*Rhizobia** | 0.2295 | 0.5722 |
| **Rep*Rhizobia*Nitrogen** | 0.5885 | 0.4718 |
| **Residual** | 5.0262 | 0.4486 |

**Tests of Covariance Parameters Based on the Restricted Likelihood – SPAD 2018**

| **Label** | **DF** | **-2 Res Log Like** | **ChiSq** | **Pr > ChiSq** | **Note** |
| --- | --- | --- | --- | --- | --- |
| **Rep=0** | 1 | 1312.60 | 0.24 | 0.3113 | MI |
| **Rep*Rhizobia=0** | 1 | 1312.56 | 0.21 | 0.3245 | MI |
| **Rep*Rhizobia*Nitrogen=0** | 1 | 1319.74 | 7.39 | 0.0033 | MI |

MI: P-value based on a mixture of chi-squares

**Carbon isotope discrimination (δ^13^C, ‰) 2018**

**Covariance Parameter Estimates - δ^13^C 2018**

| **Cov Parm** | **Estimate** | **Standard Error** |
| --- | --- | --- |
| **Rep** | -0.00910 | 0.009683 |
| **Rep*Rhizobia** | -0.01717 | 0.02998 |
| **Rep*Rhizobia*Nitrogen** | 0.07475 | 0.04655 |
| **Residual** | 0.1278 | 0.01147 |

**Tests of Covariance Parameters Based on the Restricted Likelihood - δ^13^C 2018**

| **Label** | **DF** | **-2 Res Log Like** | **ChiSq** | **Pr > ChiSq** | **Note** |
| --- | --- | --- | --- | --- | --- |
| **Rep=0** | 1 | 350.36 | 3.23 | 0.0721 | DF |
| **Rep*Rhizobia=0** | 1 | 350.38 | 3.25 | 0.0715 | DF |
| **Rep*Rhizobia*Nitrogen=0** | 1 | 407.53 | 60.40 | <.0001 | DF |

DF: P-value based on a chi-square with DF degrees of freedom.

Because estimation is unconstrained, likelihood-ratio tests are conducted as two-sided tests.
